# Ethnobotanical study of traditional medicinal plants used by the local people in Habru District, North Wollo Zone, Ethiopia

**DOI:** 10.1186/s13002-023-00644-x

**Published:** 2024-01-04

**Authors:** Mulugeta Alemu, Zemede Asfaw, Ermias Lulekal, Bikila Warkineh, Asfaw Debella, Bihonegn Sisay, Eyob Debebe

**Affiliations:** 1 Department of Plant Biology and Biodiversity Management, College of Natural and Computational Sciences, Addis Ababa University, Addis Ababa, Ethiopia; 2 Department of Urban Agriculture, Nefas Silk Polytechnic College, Addis Ababa, Ethiopia; 3 Armauer Hansen Research Institute, Addis Ababa, Ethiopia

**Keywords:** Biodiversity conservation, Ethnobotany, Habru District, Traditional herbal knowledge, Traditional medicinal plants

## Abstract

**Background:**

Ethiopia is a country located in the Horn of Africa, which combines richness in plant resources and cultures of human plant use. The people of Habru District of North Wollo Zone (Amhara Region, Ethiopia) have a long history of use of plant resources for various purposes including in traditional herbal remedy preparation and use. However, the district has not been adequately studied for its ethnobotanical resources and the associated knowledge. This study focused on human medicinal plants and their traditional uses in Habru District. The objective of the study was to document and analyze the plant species used by the local communities to treat human ailments along with the associated traditional knowledge and practices.

**Methodology:**

The study was carried out in Habru District from June 2021 to December 2022. Ethnobotanical data were collected using semi-structured interviews, guided field walks, 13 focus group discussions (one at the district level and 12 at the kebele/subdistrict level) and market surveys. A total of 388 informants (250 males and 138 females) were selected from all 13 kebeles within Habru District using systematic random sampling, and 42 key informants were purposively selected. Descriptive statistics, preference ranking, direct matrix ranking, informant consensus factor and fidelity level were applied for data analysis.

**Results:**

The results provide insights into the medicinal plant diversity within Habru District, where 134 plant species in 110 genera and 54 families were documented, including 2 endemics, highlighting the district’s significance in biodiversity conservation and healthcare delivery. Disease prevalence analysis showed that gastrointestinal and parasitic ailments (ICF = 0.85), febrile diseases (ICF = 0.84), and culture-related conditions exhibit high informant consensus factors. Remedy preparation involves various plant parts, predominantly leaves (47.3%), followed by roots (22.1%), fruits (7.0%), and seeds (5.8%). Freshly harvested plant parts were frequently used (58.2%), while 24.7% involved both dried and fresh parts. Oral application (47.3%) and topical use (31.8%) are the major routes of remedy administration. The marketability of medicinal plants was evident, with 16.4% of the species reported as marketable, including *Terminalia brownii* Fresen. *Myrtus communis* L., *Ruta chalepensis* L., *Olea europaea* L. subsp. *cuspidata* (Wall. & G.Don) Cif., *Allium sativum* L. and *Capsicum annuum* L. Multipurpose plants such as *Solanum somalense* Franchet. (91.3% FL), *Ocimum lamiifolium* Hochst. ex. Benth. (88.9% FL), and *Verbascum sinaiticum* Benth. (85.7% FL) exhibited notable healing potentials.

**Conclusion:**

The current study underscores the intricate relationship between the local community and medicinal plants, emphasizing the importance of biodiversity conservation and health care and acknowledging the dynamic interplay between cultural heritage and ecosystem health. The results contribute to the development of sustainable conservation strategies, healthcare practices and the preservation of traditional knowledge, and highlight the interdependence of human societies and their natural environments. Community-based conservation initiatives with active participation of local communities are desirable for the conservation and sustainable use of medicinal plant species and their habitats. Raising public awareness about the sustainable harvesting and utilization of marketed medicinal plants (e.g., *Terminalia brownii* Fresen.) that are under threat is also important to ensure their availability for future generations and contribution to socioeconomic development.

## Background

Medicinal plants are vital resources for primary health care of people around the world [1]. Approximately 80% of the global population is estimated to utilize medicinal plants for disease treatment, and in African nations, this percentage is even higher [2–4]. Ethiopia is a renowned hub for ethnomedicinal research due to its remarkable plant diversity, cultural richness and profound traditional knowledge and ancient medical practices [5, 6]. Among the twelve Vavilov centers of origin, Ethiopia exhibits an enormous diversity of domesticated crops and their wild relatives, demonstrating a vast array of plant genetic resources [7, 8]. The flora of Ethiopia is estimated to contain close to 5757 vascular plant species, with approximately 10% endemic to the flora area [9, 10]. In the healthcare system of Ethiopia, traditional medicine is widely practiced alongside modern medical approaches [11, 12]. About 80% of Ethiopian population rely on traditional medicines (TMs) for their health care, and more than 95% of the preparations are made from plants [11, 13]. However, these medicinal plants face various threatening factors, including habitat destruction, urbanization, agricultural expansion, deforestation, firewood collection, and environmental degradation [14–17].

Various ethnobotanical studies have been conducted in different parts of Ethiopia to document the extensive use of medicinal plants to treat human and livestock ailments [16–29]. Despite the crucial role of medicinal plants in Ethiopia's traditional primary health care, the geographical and cultural coverage is limitted and so are attempts to scientifically explore, document and validate the depth of the associated knowledge [17].

Similar to elsewhere in Ethiopia, people living in Habru District have also traditional practices to take care of themselves and the health of their livestock [30]. Additionally, the ecology of Habru District is characterized by highlands, middle lands, and lowlands. Due to the diverse ecological landscape, the diversity and practices of traditional MP species are expected to be more in the study area. Therefore, a comprehensive ethnobotanical study in Habru District is essential to document and analyze the traditional knowledge and practices of the local people focusing on medicinal plants for the treatment of human ailments, contributing to the utilization and preservation of biodiversity. Moreover, comparing the findings of this study with the Ethiopian ethnobotanical medicinal plant database will provide valuable insights into the regional distribution and utilization of medicinal plants, further enriching our understanding of Ethiopia's remarkable traditional plant-based healthcare system. In view of this, the present study aims to (i) collect, identify, and document medicinal plants and the associated indigenous knowledge of the local people used to treat various human ailments in the study area (ii) identify and document marketable medicinal plants used in the study area and (iii) select candidate MP species of high informant consensus and fidelity level values for antimicrobial and phytochemical analyses in our subsequent studies.

## Materials and methods

### Description of the study area

**Fig. 1:**
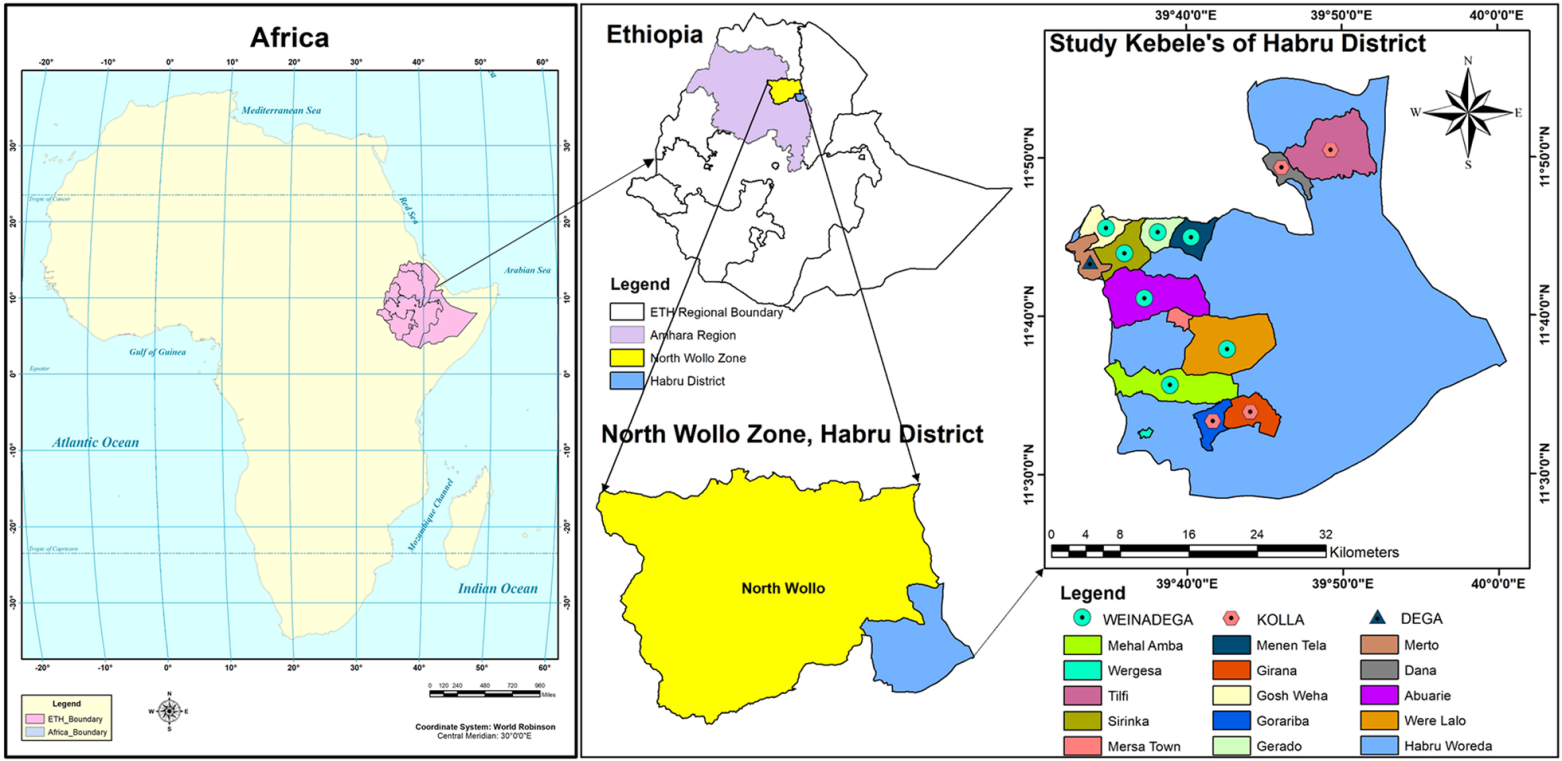
Map of Ethiopia showing the Amhara region and the location of the study district (developed using ArcGIS 10.5)

**Fig. 2:**
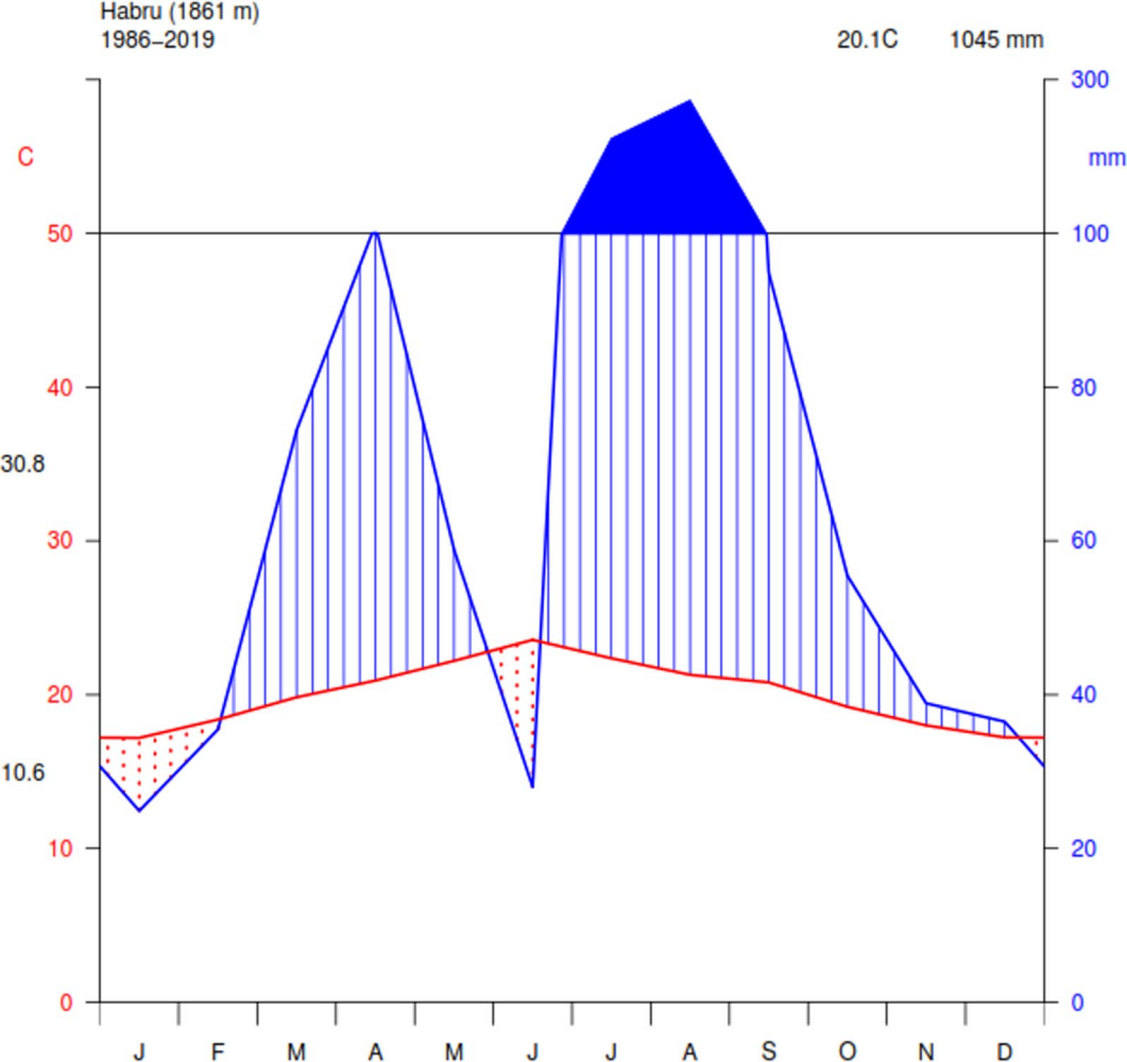
Climate diagram of the Habru District showing rainfall distribution and temperature variation from 1986 to 2019. Data source: Ethiopian National Meteorology Agency (ENMA**)**

The study focuses on the ethnobotany of medicinal plants in the Habru District, situated in the North Wollo Zone of the Amhara Region, Ethiopia (Fig. 1). Habru District has 36 rural kebeles (subdistricts) and three urban administrations, with a total area of 1350.4322 km^2^. According to the Habru District Agriculture and Rural Development Office 2019 report, Mersa town is the center of the District, which is 88 km north of Dessie town and 491 km north of Addis Ababa, the Ethiopian capital, 406 km west of Bahirdar (Amhara Region’s capital), 30 km northeast of Woldiya (North Wollo Zone capital) [30]. The grid references for Harbu District come within 11°35′0″–11°55′0″ N and 39°30′10″–40°10′0″ E with an altitudinal range of 1430–2800 masl. It is bordered to the south by the Mille River, separating it from the South Wollo Zone. To the west lies Gubalafto District, while the Alawuha River forms the northern boundary, separating North Wollo from Kobo woreda. The eastern border is defined by the Afar Region. The weather condition of Habru District is characterized by the cold climate which is locally known as dega (temperate) and covers 3.5% of the total area of the district, the warmest, which is locally known as kolla (tropical) and covers 56.5% and medium, which is locally known as weinadega (subtropical) and covers 40% [30]. Rainfall data from 1986 to 2019 were obtained from the Ethiopian National Meteorology Agency (ENMA) Sirinka station, the mean annual temperature of the study area is 20.1 °C (Fig. 2). This corresponds to the monthly minimum and maximum temperatures of 10.6 °C and 30.8 °C, respectively [31]. Habru District has a population of 192,742, with a majority residing in rural areas [30]. The population density is 155.46 persons per square kilometer, higher than the zone average. The inhabitants mainly practice Muslim (76.85%) and Ethiopian Orthodox Christianity (22.95%) [30]. The economic activities in Habru District are centered around large commercial farms focused on livestock rearing and agro-processing, particularly in the Girana kebele. The district benefits from market access facilitated by the main road connecting it to Addis Ababa, and local products are sold in nearby markets such as Mersa and Dessie town.

### Reconnaissance survey and site selection

Before the commencement of the reconnaissance survey, an official letter was received from the Department of Plant Biology and Biodiversity Management (DPBBM) at Addis Ababa University. Additionally, verbal informed consent was obtained from each participant involved in the study during the designated period. The reconnaissance survey took place between June 14, 2021, and July 02, 2021, within the confines of Habru District. Its purpose was to develop an initial understanding of the agro-ecological characteristics of the region, the prevailing vegetation status, the local population's indigenous knowledge regarding plant applications for various uses, and an assessment of accessibility and other pertinent environmental conditions. To select study kebeles, the purposive sampling method was employed, with a focus on identifying kebeles with better vegetation cover and a well-known history of utilizing medicinal plants. These kebeles were also deemed potential sites for home gardening activities. In addition, the selection of study sites was underpinned by prior insights gathered from participants in focus group discussions (FGDs), community leaders, venerable elders, traditional healers, and healthcare personnel within the locality. As a result, a total of 13 study kebeles were chosen, representing 35% of the entire kebele count within Habru District. These selected study kebeles are enumerated as follows: Worgesa, Gosh Weha, Sirinka, Gerado, Girana, Dana, Abuarie, Tilfi, Menen Tela, Merto, Gorariba, Mehal Amba, and Were Lalo.

### Sample size determination and selection of informants

The determination of the informant sample size adhered to the methodology outlined in [32] as cited in [33]. Accordingly, the sample size (n) was calculated using the formula:

$$n = \frac{N}{{1 + N \left( e \right)^{2} }}$$

where *n* = sample size to be used for this research, *N* = total number of households (HH) in all subdistricts, *e* = maximum variability or margin of error 5% (0.05), 1 = the probability of the event occurring. To establish the number of households to be interviewed within each kebele, the following formula was applied:

$$A = \frac{B \times n}{N}$$

where *A* = sample size to be used in a given kebele, *B* = total number of households (HH) found in a given kebele, *n* = total sample size to be used for this research, and *N* = total number of households (HH) in all subdistricts.

The research encompassed a total of 388 informants (250 males and 138 females) from all 13 kebeles within Habru District (Table 1). The selection of these informants followed systematic random and purposive sampling methods, including peer recommendations, as described by [34]. Nominations of traditional herbalists to participate as key respondents were collected from community members, elderly people and knowledgeable inhabitants as the methods recommended by [35, 36] and used to identify 42 key informants (34 men and 8 women) among the inhabitants, whereas general informants were systematically sampled during random visits made to houses in the study kebeles.

**Table 1:** Description of kebeles (subdistricts) of data collection within the study area, highlighting key geographical and demographical attributes

Study kebele/village name	GPS coordinates		Elevation (m)	Ecology	Total number of HH	Number of interviewees	Gender		Age		Occupation		Religion	
	Latitude	Longitude					M	F	YM	El	Li	Il	Ch	Mu
Wergesa	11°32′37.38″ N	39°37′20.53″ E	1848	SuT	1600	48	24	24	18	30	19	29	23	25
Gosh Weha	11°45′17.86″ N	39°34′45.29″ E	2316	SuT	663	20	13	7	5	15	7	13	12	8
Sirinka	11°44′1.77″ N	39°35′59.59″ E	2111	SuT	780	24	18	6	7	17	5	19	21	3
Gerado	11°45′5.36″ N	39°38′2.04″ E	1817	SuT	674	20	8	12	6	14	5	15	11	9
Girana	11°34′0.43″ N	39°44′8.96″ E	1425	Tro	1301	39	22	17	15	24	8	31	4	35
Dana	11°49′28.33″ N	39°46′17.45″ E	1545	Tro	734	22	16	6	6	16	11	11	0	22
Abuarie	11°41′14.17″ N	39°36′49.34″ E	1811	SuT	1707	51	36	15	9	42	17	34	41	10
Tilfi	11°50′31.75″ N	39°49′21.32″ E	1534	Tro	729	22	19	3	11	11	3	19	0	22
Menen Tela	11°44′52.20″ N	39°40′29.71″ E	1749	SuT	585	18	11	7	1	17	3	15	10	8
Merto	11°43′26.68″ N	39°33′41.16″ E	2589	Tem	1000	30	20	10	11	19	7	23	22	8
Gorariba	11°33′7.89″ N	39°41′39.22″ E	1547	Tro	514	16	13	3	2	14	6	10	2	14
Mehal Amba	11°35′36.91″ N	39°39′5.25″ E	1645	SuT	1966	59	35	24	22	37	23	36	9	50
Were Lalo	11°38′11.28″ N	39°42′41.59″ E	1449	SuT	632	19	15	4	3	16	5	14	2	17
Total	12,885	388	250	138	116	272	119	269	157	231				

Key: Household, HH; Ecology (Subtropical, SuT; Tropical, Tro and Temperate, Tem); Gender (Male, M and Female, F); Occupation (Literate, Li denotes interviewees who have completed at least primary education and Illitrate, Il); Age categories include young to middle-aged, YM (19–39 years) and elderly, El (40–98 years). Religion (Christian, Ch encompasses orthodox, catholic, and protestant and Muslim, Mu). Regarding ethnicity and language, all interviewees belong to the Amhara ethnic group and speak the Amharic language

#### Data collection

The ethnobotanical data collection encompassed three distinct field trips carried out between June 14, 2021, and December 14, 2022. Qualitative and quantitative ethnobotanical data were collected from informants through a pre-prepared semi-structured interview method, as described by [34–36]. Notably, field notes were meticulously recorded, safeguarding the discreet knowledge of the local community [34]. To ensure confidentiality, necessary ethical clearance was secured by briefing the informants, and the fieldwork adhered to Bennett’s Golden Rules [37]. In the broader context of this study, techniques such as group discussions, semi-structured interviews, guided field walks, market surveys, preference ranking, and direct matrix ranking were employed, as recommended by [34].

#### Focus group discussion and semi-structured interview

Semi-structured questionnaires were employed to facilitate discussions and interviews with informants, enabling the collection of pertinent information aligned with the research objectives. The methods, procedures, and techniques followed those recommended by standard ethnobotany sources [35]. Additionally, a total of 13 focus group discussions were carried out, one at the district level and 12 at selected kebeles of the district. In each focus group discussion key informants, traditional healers, elders, kebele and district administrative officials from natural resource and forest protection offices, and agricultural and rural development offices were involved to amplify insights into medicinal plant knowledge at the community level and to corroborate information obtained through semi-structured interviews [34]. Informants were interviewed separately in the local Amharic language, addressing queries about their general information. Furthermore, interviewees provided local names of medicinal plants, detailed information about treated ailments, species habitats, seasonality, marketability, plant parts used, condition of those parts (fresh or dried), preparation methods, dosage instructions, and routes of administration for remedies [34, 36]. Moreover, informants were asked about other (non-medicinal) uses of the medicinal plant species they mentioned to identify the overall use values and use diversities of species in the manner recommended by [34–36].

#### Guided field walk

Field observations were facilitated by the collaboration of local guides, traditional healers, district experts, and participating informants, ensuring the comprehensive acquisition of indispensable data within the study area. Through the integration of both etic and emic categorizations, invaluable insights were garnered, shedding light on the categorization of medicinal plants, plant communities, landscapes, and soil types. Additionally, the pivotal contribution of traditional healers during the guided field walk extended beyond facilitating the process; they actively participated in identifying encountered medicinal plants by providing their vernacular names, medicinal use, parts used, preparation methods, dosages, and methods of application. The collection of voucher specimens during the guided field walk was supported by digital photographs of both freshly gathered and pressed dry voucher specimens. This specimen collection endeavor encompassed diverse environments, including the wild, home gardens, crop fields and margins and local markets.

#### Market survey

Market surveys were carried out to document the medicinal plants available in local markets of the study area and gather insights into the market values of plants. This method proved instrumental in investigating the availability, pricing, and unit measurement of marketable medicinal plants, aiding the preservation of high-value medicinal plant species. To this end, four local markets namely Mersa, Mehal Amba, Wergesa and Girana were visited, and semi-structured interviews were conducted with drug vendors at the markets. A total of 18 informants (12 vendors and 6 user of MPs) were interviewed, with 7 men and 11 women, representing participants from all four local market areas of the study. These interviews aimed to obtain general information on the multipurpose roles and marketability of medicinal plant species, and their plant-derived products sold on markets were recorded.

#### Voucher specimen collection and identification

Voucher specimens of reported medicinal plants were collected and the preliminary identification was executed using manuals in the field and they were pressed, dried, deep frozen, and identified. The identification was performed using the keys from published volumes of the Flora of Ethiopia and Eritrea, followed by comparisons with authenticated specimens in the National Herbarium (ETH) of Addis Ababa University, and ultimately confirmed by taxonomic experts at Addis Ababa University. The identified specimens with voucher numbers, families, species, and vernacular names, dates and sites of collection were recorded and deposited at the National Herbarium (ETH) at AAU.

### Data analysis

For this study, a combination of qualitative and quantitative ethnobotanical analytical tools was employed, adhering to the relevant methodologies proposed by [34, 36]. Ethnobotanical data were entered into an Excel spreadsheet, version 2013, and subjected to comprehensive analysis using descriptive statistics. This facilitated the identification of the most frequently employed multipurpose plants within the study area. To elucidate the proportions of different plant species, growth forms, sources of collection, plant parts used, methods of preparation, and related aspects, a range of ethnobotanical scoring and ranking techniques, percentage frequency methodologies, and inferential statistics were employed. The findings were subsequently presented through graphs, charts, tables, and textual explanations.

The preference ranking (PR) technique involved the systematic arrangement of lists or groups of plants or resources based on a designated criterion [34]. In this study, key informants were requested to assign values or scores for ranking the most preferred plant for treating the most commonly reported human ailments within the study area. Each informant ranked the items according to individual preference or perceived importance within the community. The items were assigned numerical values, with the most vital receiving the highest number, descending in value as the significance of the items diminished. The least preferred or important item was denoted by the lowest value, which was "1."

Direct matrix ranking (DMR) was conducted in line with the method described by [34], aiming to compare the multipurpose use of medicinal plants. The process involved soliciting informants to sequentially order a given set of items according to specific attributes. This procedure was applied to ten multipurpose medicinal plants and the five most commonly cited factors perceived as threatening by key informants, following [36]. Each informant provided use values ranging from 5 for “excellent” to 0 for "not used."

The informant consensus value was calculated based on the free listing data collected during interviews, which were then summarized in tabular form [38]. The level of agreement between information provided by various informants was assessed using the Informants' Consensus Factor (ICF) formula [39]:

$${\text{ICF}} = \frac{{N_{ur} - Nt}}{{\left( {N_{ur} - 1} \right)}}$$

where *N*_ur,_ is the number of use reports from informants for a particular plant usage category, and *N*_t_ is the number of species that are used for the plant usage category for all informants. Values range between 0 and 1, where 1 indicates the highest level of informant consent. As described by [40], medicinal tradition is viewed as well defined if a high degree of consensus is recorded. This means that a high value indicates that relatively few taxa are used by a larger proportion of the healers, while a low value indicates that informants disagree on the taxa to be used in the treatment within a category of illness.

Fidelity level (FL) has been employed to quantify the importance of a given species for a particular purpose in a given cultural group [41] cited in [35]. It is the percentage of informants claiming the use of a certain plant species for the same major purpose, and FL was calculated for the most frequently reported diseases or ailments as follows:

$$FL \left( \% \right) = \left( \frac{Np}{N} \right) \times 100$$

where *Np* is the number of informants that claim the use of plant species to treat a particular disease; and *N* is the number of informants that use the plants as a medicine to treat any given disease.

### Knowledge difference according to demographic characteristics of informants

For this study, ethnobotanical knowledge exhibits distinct variations based on the diverse demographic characteristics of the informants. Male general informants, constituting 62.4% of the participants, reported about the use of plants as medicine in the study area, while the key informants were predominantly masculine (81%). The study cohort encompassed a wide age range, with 29.9% falling into the young-to-middle-aged category (19–39 years old) and 70.1% comprising the elderly individuals (40–98 years). Notably, 30.7% of the informants displayed literacy, while the majority remained illiterate (69.3%). Regarding marital status, the majority of informants were married (85.3%), followed by divorced (7.2%), widowed (4.4%), and single (3.1%) household representatives. This intricate demographic mosaic contributes to the research's holistic scope, ensuring comprehensive representation across gender, age, literacy levels, and marital statuses.

## Results

### Diversity of reported medicinal plants in Habru District

**Fig. 3:**
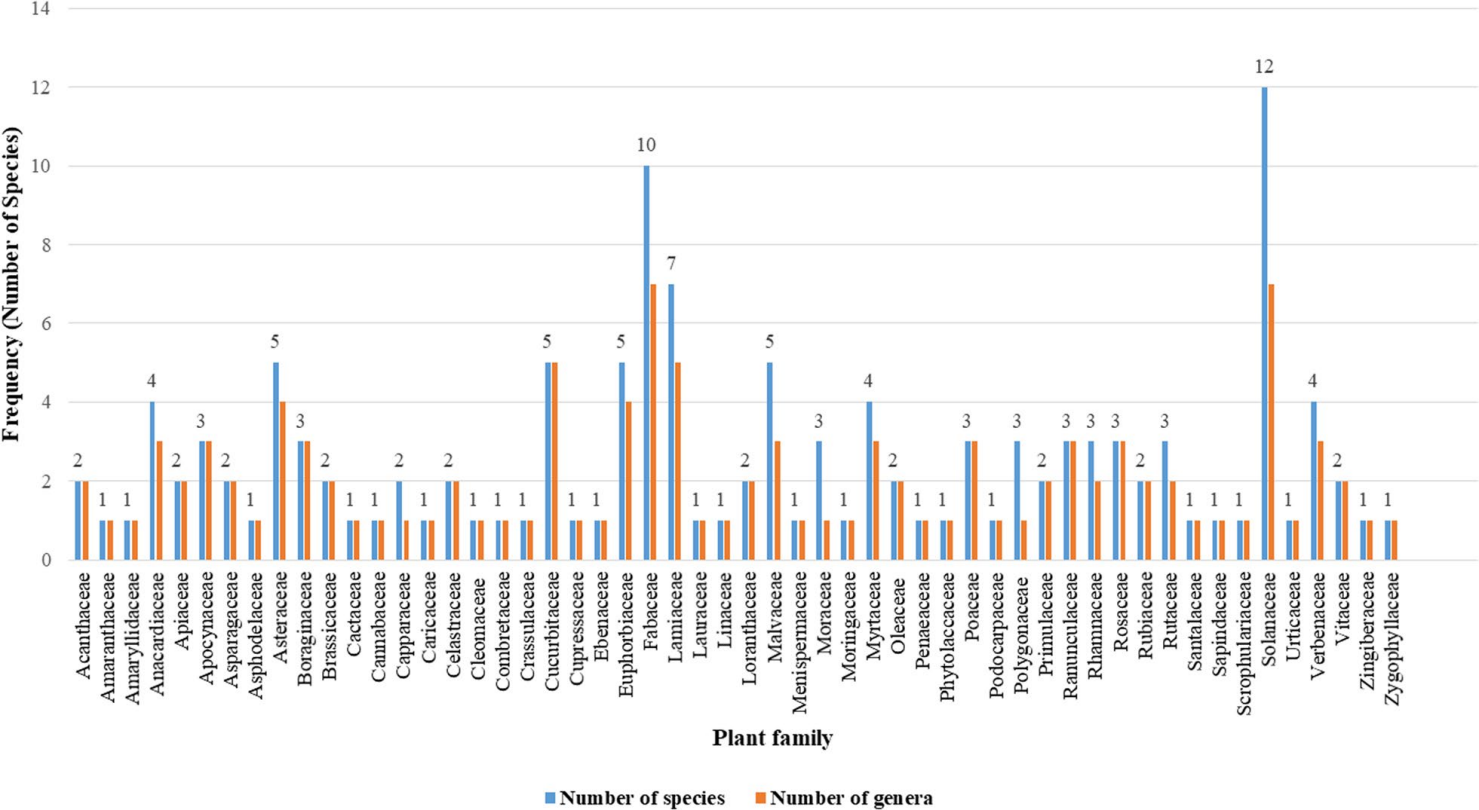
Distribution of reported medicinal plant species in Habru District across different families

**Fig. 4:**
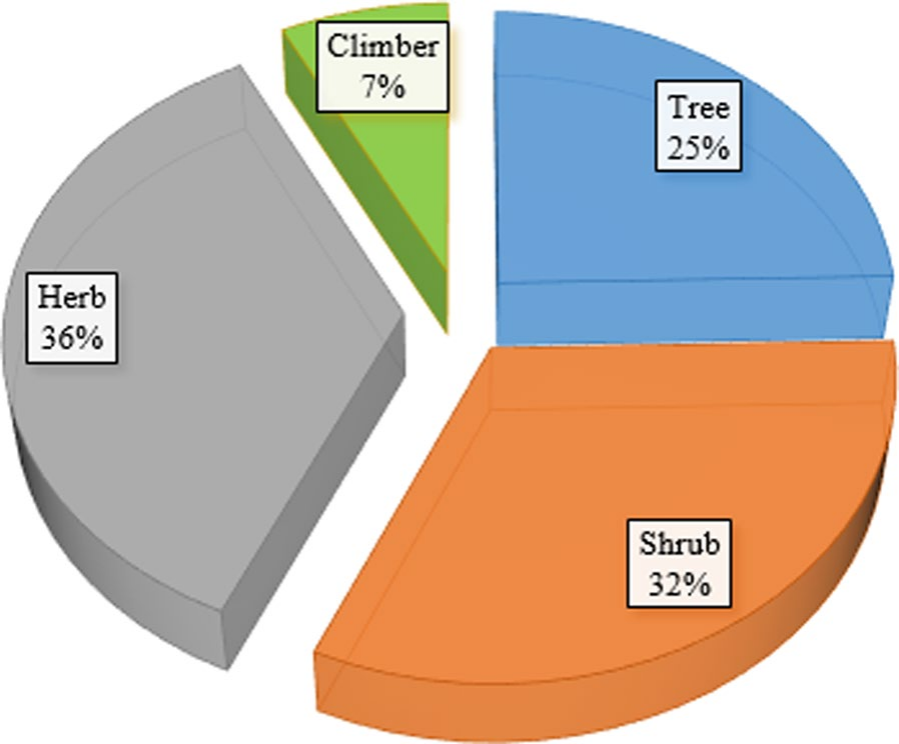
Growth form of medicinal plants collected in Habru District

A total of 134 medicinal plant species belonging to 110 genera and 54 botanical families consisting of 132 angiosperms and 2 gymnosperms were reported to be used for treating human ailments in Habru District (Fig. 3 and Table 2). Of these plants, herbs took the highest proportion (36%) whereas climbers took the least proportion (Fig. 4). The family Solanaceae was represented by the highest number of species (12 species, 9%) followed by Fabaceae (10 species, 7.5%) and Lamiaceae (7 species, 5.2%). Asteraceae, Cucurbitaceae, Euphorbiaceae, and Malvaceae each contributed 5 species (3.7% of the total). In contrast, Anacardiaceae, Myrtaceae, and Verbenaceae each represented with 4 species. The top 15 medicinal plant species identified in the study area based on the use citation, compared to similar studies conducted in another region of Ethiopia are presented in Table 3.

**Table 2:** List of medicinal plants used for the treatment of human ailments in Habru District, Amhara Region, Ethiopia: scientific name; family; local name; growth form; ailment treated; plant parts used; condition of plant part uses; methods of preparation and application, route of administration, plant part mixed with and voucher number

Scientific names	Local name (Amharic)	Family name	GF	AT	PPU	CPU	MPAP	RA	UC	VS number
*Barleria eranthemoides* R.Br.ex C.B.Clarke	Yeset Af	Acanthaceae	H	Boils	L	F	3	De	2	MA13
				Diarrhea	R	F	1	O	1	
				Wound	R	F	2	De	3	
*Justicia schimperiana* (Hochst. ex Nees) T.Anderson	Sensel, Simiza	Acanthaceae	S	Febrile disease	L	F	10	ODN	6	MA62
				Typhoid	L	F	16	O	5	
				Malaria	L	F	16	O	2	
				Liver problem	L	F	5	O	2	
*Agave sisalana* Perrine	Chiret, Kacha	Asparagaceae	S	Wound	Lat	F	4	De	2	MA06
*Allium sativum* L. *Aloe macrocarpa* Tod	Nech Shnkurt	Amaryllidaceae	H	Atopic eczema	Bu	F	4	De	3	MA08
				Asthma	Bu	F	4	De	3	
				Common cold	Bu	DF	11	O	6	
				Dandruff	Bu	DF	1	O	2	
				Pneumonia	Bu	F	4	De	1	
				Coughing	Bu	DF	1	O	5	
				Malaria	Bu	DF	11	O	1	
	Iret/Iret tafa	Asphodelaceae	H	Gastritis	L	F	11	O	2	MA09
				Stomachache	L	F	11	O	2	
				Malaria	Lat	F	11	O	1	
				Wound	Lat	F	4	De	2	
*Achyranthes aspera* L.	Telenj	Amaranthaceae	H	Nasal infection	R	F	7	Na	4	MA05
				Minor bleeding	L	F	5	O	2	
				Wound	L	F	5	O	2	
*Searsia retinorrhoea*(Steud. ex Oliv.) Moffett	Talo/Talo Embis	Anacardiaceae	T	Stomachache	R	D	5	O	6	MA96
				Tonsillitis	L	F	5,11	O	10	
*Schinus molle* L.	Kundo-berberie	Anacardiaceae	T	Common cold	L	F	8	Na	3	MA107
				Jaundices	Fr	D	5	O	2	
*Searsia pyroides*(Burch.) Moffett	Yeahya Talo, Yeregna Qolo	Anacardiaceae	S	Wound	L	D	3	De	1	MA97
*Mangifera indica* L.	Mango	Anacardiaceae	T	Impotency	Fr	F	9	O	2	MA72
*Ferula communis* L.	Dog	Apiaceae	H	Impotency	R	D	13	O	2	MA49
*Foeniculum vulgare* Mill	Ensilal	Apiaceae	H	Headache	R	F	5	O	3	MA53
				Stomachache	R	D	5	O	2	
*Carissa spinarum* L.	Agam	Apocynaceae	S	Diarrhea	R	DF	5	O	2	MA22
				Constipation	R	DF	5	O	1	
				Evil spirit	R	DF	8	Na	2	
				Headache	L	F	4	De	1	
				Snake bite	L	F	9	O	3	
*Calotropis procera* (Aiton) W.T.Aiton	Tobia	Apocynaceae	S	Atopic eczema	Lat	F	4	De	2	MA15
				Hemorrhoid	Lat	F	4	De	3	
				Hemorrhoid	R	F	4	An	4	
*Periploca linearifolia* Quart.-Dill. & A. Rich	Moider	Apocynaceae	C	Diabetes	LR	F	1	O	1	MA111
*Asparagus africanus* Lam	Yeset Kest, Kestanicha	Asparagaceae	H	Skin diseases	R	F	12	De	1	MA133
*Bidens pilosa* L.	Yeseytan Merfe	Asteraceae	H	Snake bite	L	DF	5	O	2	MA14
				Wound	L	F	3	De	3	
				Evil spirit	Wp	DF	7	ODN	4	
*Artemisia absinthium* L.	Arity, Natra	Asteraceae	H	Evil spirit	L	F	8	Na	2	MA10
*Guizotia abyssinica* (L.f.) Cass	Nug	Asteraceae	H	Gastritis	Se	D	9	O	2	MA57
*Gymnanthemum amygdalinum*(Delile) Sch.Bip	Girawa	Asteraceae	T	Stomachache	L	F	5	O	2	MA122
				Giardia	L	DF	15	O	2	
				Wound	L	D	2	De	1	
*Artemisia abyssinica* Sch.Bip. ex Oliv. & Hiern	Chikugn	Asteraceae	H	Evil Eyes	Wp	F	8	Na	3	MA11
*Balanites aegyptiaca* (L.) Delile	Bedena	Zygophyllaceae	T	Stomachache	L	F	5	O	1	MA12
				Dandruff	L	F	4	De	2	
*Ehretia cymosa* Thonn	Wulaga	Boraginaceae	T	Evil spirit	Hp	F	7	Na	28	MA42
*Cordia africana* Lam	Wanza	Boraginaceae	T	Atopic eczema	L	F	4	De	1	MA34
*Cynoglossum coeruleum* Hochst. ex A.DC	Fikru-tena, Chigogot	Boraginaceae	H	Febrile disease	L	F	7	NA	1	MA132
*Lepidium sativum* L.	Feto	Brassicaceae	H	Tonsillitis	Se	DF	5	O	3	MA67
				Gingivitis	Se	DF	6	O	2	
				Fever	Se	DF	1	O	1	
				Wound	Se	DF	3	De	2	
				Boils due to bacteria S. aures	Se	DF	3	De	2	
*Sisymbrium officinale* (L.) Scop	Yewef Gomen	Brassicaceae	H	Skin diseases	L	F	3	De	2	MA109
*Opuntia ficus-indica* (L.) Mill	Beles, Qulqual	Cactaceae	S	Coughing	Hp	F	5	O	2	MA86
*Capparis tomentosa* Lam	Gimero	Capparaceae	S	Evil spirit	R	DF	7	Na	3	MA18
				Evil Eyes	R	DF	7	Na	2	
*Cleome gynandra* L.	Abethoye	Cleomaceae	H	Wound	L	F	4	De	5	MA31
				Ear infection	L	F	2	Au	10	
				Evil spirit	L	DF	11	O	12	
*Capparis decidua* (Forssk.) Edgew	Kontir	Capparaceae	S	Anthrax	Se	D	5	O	2	MA17
*Carica papaya* L.	Papaya	Caricaceae	T	Gastritis	Fr	F	9	O	3	MA21
*Catha edulis* (Vahl) Endl	Chat	Celastraceae	H	Intestinal parasite	L	F	11	O	1	MA23
*Gymnosporia senegalensis*(Lam.) Loes	Atat	Celastraceae	S	Eye disease	Fl	F	3	Op	1	MA73
				Evil spirit	R	D	5	O	2	
				Sexual						
Impotency	SB	D	9	O	3					
*Terminalia brownii* Fresen	Wyba	Combretaceae	T	Wound	SB	D	7	Na	1	MA116
*Kalanchoe petitiana* A.Rich	Endahula	Crassulaceae	H	Tonsillitis	R	F	8	Na	4	MA63
				Stomachache	R	F	9	O	6	
				Swelling	L	F	12	De	3	
*Cucumis ficifolius*A.Rich	Yemidir Embway	Cucurbitaceae	H	Evil spirit	L	DF	7	Na	1	MA36
				Rabies	R	D	1	O	2	
				Stomachache	R	F	5	O	2	
				Wound	L	F	3	De	4	
				Febrile disease	L	F	10	ODN	3	
*Zehneria scabra* (Linn.f.) Sond	Hareg resa/Etse Sabieq	Cucurbitaceae	H	Febrile disease	L	F	10	ODN	3	MA125
				Wound	L	F	4	De	2	
				Liver problem	L	F	5	O	1	
*Cucurbita pepo* L.	Duba	Cucurbitaceae	C	Tap worm	Se	D	9	O	1	MA37
				Ear lesion	Fl	F	2	Au	2	
*Lagenaria siceraria* (Molina) Standley	Qil	Cucurbitaceae	C	Liver problem	L	F	5	O	1	MA64
*Momordica foetida* Schumach	Yekura Hareg	Cucurbitaceae	C	Wound	L	F	3	De	2	MA75
				Coughing	Wp	DF	7	Na	3	
*Juniperus procera* Hochst. ex Endl	Yehabesha Tsid	Cupressaceae	T	Fire burn	SB	D	2	De	2	MA61
*Euclea racemosa* L.	Dedeho	Ebenaceae	S	Wound	L	F	3	De	19	MA46
*Croton macrostachyus* Hochst. ex Delile	Bisana	Euphorbiaceae	T	Atopic eczema	L	F	4	De	4	MA35
				Liver problem	B	DF	5	O	2	
				Stomachache	B	D	15	O	2	
				Gonorrhea	B	F	5	O	3	
				Malaria	Fr	F	13	O	1	
				Chronic skin diseases	L	F	4	De	4	
				Scabies	L	F	4	De	4	
				Wound	L	F	4	De	3	
				Minor bleeding	L	F	4	De	2	
				Febrile disease	L	DF	7	Na	1	
*Euphorbia tirucalli* L.	Kinchibt	Euphorbiaceae	S	Wound	Lat	F	4	De	3	MA48
*Ricinus communis* L.	Gulo	Euphorbiaceae	S	Stomachache	R	DF	7	ODN	2	MA98
				Ear mites	L	F	3	Au	3	
				Hemorrhoid	L	F	4	De	1	
				Scabies	Fr	F	4	O	2	
				Evil spirit	R	F	5	O	1	
*Euphorbia abyssinica* J.F.Gmel	Kulkual	Euphorbiaceae	S	Hemorrhoid	Lat	F	4	De	1	MA47
				Take out spine	Lat	F	4	De	2	
*Tragia brevipes* Pax	Aleblabit, Ablalit	Euphorbiaceae	H	Stomachache	L	F	5	O	1	MA134
*Vachellia seyal*(Delile) P.J.H.Hurter	Wacho Grar	Fabaceae	T	Wound	L	F	3	De	2	MA03
*Trigonella foenum-graecum* L.	Abish	Fabaceae	H	Gastritis	Se	D	5	O	1	MA118
*Pterolobium stellatum* (Forssk.) Brenan	Kentafa	Fabaceae	S	Asthma	B	D	2	Na	2	MA94
*Cicer arietinum* L.	Shimbira	Fabaceae	H	Malaria	Se	D	11	O	1	MA26
*Millettia ferruginea* (Hochst.) Bak	Birbira	Fabaceae	T	Skin infection	Fr	D	4	De	1	MA74
*Senegalia senegal* (L.) Britton	Sibansa Grar	Fabaceae	T	Impotency	R	DF	9	O	3	MA02
				Eye infection	B	DF	3	Op	2	
*Vachellia tortilis*(Forssk.) Galasso & Banfi	Korera	Fabaceae	T	Wound	L	F	4	De	4	MA04
*Dichrostachys cinerea* (L.) Wight & Arn	Awrarise/Ader/Gorgoro	Fabaceae	S	Skin diseases	R	DF	2	De	2	MA40
*Vachellia oerfota*(Forssk.) Kyal. & Boatwr	Ajo, Chelegama	Fabaceae	T	Coughing	B	DF	5	O	1	MA01
				Scabies	L	F	3	De	2	
*Calpurnia aurea* (Ait.) Benth	Digita	Fabaceae	S	Diarrhea	R	DF	5	O	12	MA16
				Snake bite	R	DF	5	O	2	
				Excessive bleeding after birth	R	DF	5	O	2	
*Ocimum lamiifolium* Hochst. ex. Benth	Dama Kessie	Lamiaceae	H	Febrile disease	L	F	4	De	24	MA82
*Ocimum basilicum* L.	Besobila	Lamiaceae	H	Swelling	Wp	DF	4	De	1	MA81
				Stomachache	L	F	5	O	1	
*Ocimum gratissimum* (L.)subsp.*gratissimum*	Damakasse, Kesedama	Lamiaceae	S	Malaria	Wp	DF	4	De	3	MA83
*Salvia nilotica* Juss. ex Jacq	Hulgeb	Lamiaceae	H	Wound	L	F	4	De	16	MA07
*Rotheca myricoides*(Hochst.) Steane & Mabb	Misiroch	Lamiaceae	H	Wound	L	DF	4	De	3	MA32
*Leonotis ocymifolia* (Burml. f.) Iwarsson	Geram Tinjut, Eraskimir	Lamiaceae	S	Swelling	L	D	5	O	1	MA130
*Ajuga integrifolia* Buch.-Ham. Ex D. Don	Armagusa/Dem Akurt	Lamiaceae	H	Tonsillitis	L	F	5	O	1	MA129
*Persea americana* Mill	Avocado	Lauraceae	T	Dandruff	Fr	F	4	De	2	MA88
*Linum usitatissimum* L.	Telba	Linaceae	H	Coughing	Se	D	7	Na	5	MA68
				Stomachache	Se	D	9	O	13	
*Loranthella deflersii*(Tiegh.) S.Blanco & C.E.Wetzel	Yebedena Tegedra	Loranthaceae	H	Dactylitis	L	F	3	De	1	MA85
*Sida schimperiana* Hochst. ex A. Rich	Chifrig	Malvaceae	S	Eye disease	L	F	2	Op	2	MA108
*Malva verticillata* L.	Lut/Adguar	Malvaceae	C	Vomiting	R	F	5	O	1	MA71
*Stephania abyssinica* (Quart.-Dill. & A.Rich.) Walp	Yeayit Joro/Etse Eyesus	Menispermaceae	C	Pneumonia	L	F	5	O	2	MA115
*Ficus sur* Forssk	Sholla	Moraceae	T	Skin diseases	LR	D	2	De	2	MA51
*Ficus vasta* Forssk	Warka	Moraceae	T	Stomachache	R	F	5	O	1	MA52
*Ficus carica* L.	Beles	Moraceae	T	Wound	Lat	F	4	O	1	MA50
*Moringa oleifera* Lam	Shiferaw/Moringa	Moringaceae	T	Febrile disease	L	F	5	O	2	MA76
*Myrsine africana* L.	Kechemo	Primulaceae	S	Stomachache	LR	DF	14	O	3	MA77
*Embelia schimperi* Vatke	Enkoko	Primulaceae	S	Tap worm	Fr	D	13	O	1	MA43
*Eucalyptus globulus* Labill	Nech Bahir Zaf	Myrtaceae	T	Common cold	L	F	5	Na	8	MA45
				Coughing	L	F	5	Na	6	
				Pneumonia	L	F	5	Na	2	
				Gout	L	F	10	O	1	
				Headache	L	F	5	Na	3	
				Febrile disease	L	F	10	Na	4	
*Myrtus communis* L.	Ades	Myrtaceae	S	Scabies	L	D	2	De	2	MA78
*Psidium guajava* L.	Zeyetun	Myrtaceae	T	Gastritis	Fr	F	9	O	4	MA93
*Eucalyptus camaldulensis* Dehnh	Key Bahirzaf	Myrtaceae	T	Toothache	L	F	6	O	1	MA44
*Jasminum abyssinicum* Hochets. Ex DC	Tenbelel	Oleaceae	C	Eye disease	L	F	2	Op	7	MA60
				Common cold	R	D	7	Na	10	
*Olea europaea* L. subsp. *cuspidata* (Wall. & G.Don) Cif	Weira	Oleaceae	T	Dandruff	R	DF	2	De	12	MA84
				Stomachache	L	F	5	O	7	
*Olinia rochetiana* A. Juss	Tfie	Penaeaceae	S	Atopic eczema	L	D	3	De	1	MA131
*Phytolacca dodecandra* L'Hér	Endod	Phytolaccaceae	S	Rabies	R	DF	5	O	3	MA90
				Malaria	R	F	5	O	4	
*Saccharum officinarum* L.	Shenkora Ageda	Poaceae	T	Coughing	SB	F	9	De	6	MA106
*Cymbopogon martini* (Roxb.) Will.Watson	Teje Sar	Poaceae	H	Eye infection	L	F	3	Op	4	MA38
*Hordeum vulgare* L.	Gebs	Poaceae	H	Weight gain	SB	D	5	O	1	MA58
*Afrocarpus falcatus*(Thunb.) C.N.Page	Zigba	Podocarpaceae	T	Asthma	R	D	5	O	3	MA91
				Evil spirit	R	D	7	Na	2	
				Bone fracture	R	F	3	De	4	
				Abdominal pain	B	D	5	O	2	
*Rumex nervosus* Vahl	Embacho	Polygonaceae	S	Warts	L	DF	12	De	3	MA104
				Wound	L	DF	3	De	13	
				Fire burn	R	DF	3	Na	4	
*Rumex nepalensis* Spreng	Kitel Rejim; Tult	Polygonaceae	H	Over blood flow after birth	L	F	17	NULL	2	MA103
				Dandruff	R	F	2	De	3	
				Stomachache	R	F	5	O	1	
				Wound	L	F	3	De	1	
*Rumex abyssinicus* Jacq	Mekmeko	Polygonaceae	H	Intestinal parasite	R	D	15	O	2	MA102
*Thalictrum rhynchocarpum* Quart.-Dill. & A.Rich	Sire-Bizu	Ranunculaceae	H	Stomachache	R	F	5	O	1	MA117
*Clematis simensis* Fresen	Nech Yeazo-hareg	Ranunculaceae	C	Swelling	L	F	5	O	2	MA30
*Nigella sativa* L.	Tikur Azmud	Ranunculaceae	H	Stomachache	Se	D	5	O	3	MA80
*Ziziphus spina-christi* (L.) Desf	Kurkura, Geba	Rhamnaceae	T	Gonorrhea	R	DF	13	O	4	MA128
				Dandruff	L	F	4	De	2	
*Ziziphus mauritiana* Lam	Kurkura	Rhamnaceae	T	Breast cancer	L	F	5	O	2	MA127
*Rhamnus prinoides* L'Hér	Gesho	Rhamnaceae	S	Tonsillitis	L	F	11	O	2	MA95
*Rosa abyssinica* R.Br. ex Lindl	Kega	Rosaceae	S	Tap worm	Fr	F	9	O	2	MA99
*Prunus africana* (Hook.f.) Kalkman	Tiqur Inchet	Rosaceae	T	Urinary disorders	SB	D	5	O	2	MA92
*Rubus fruticosus* L.	Enjori	Rosaceae	S	Gastritis	L	D	5	O	3	MA101
*Rubia cordifolia* L.	Mencherer	Rubiaceae	H	Coughing	L	DF	15	O	3	MA100
				Bone fracture	L	F	3	De	1	
				Stomachache	L	F	5	O	2	
*Coffea arabica* L.	Bunna	Rubiaceae	S	Asthma	Se	D	1	O	1	MA33
*Citrus aurantiifolia* (Christm.) Swingle	Lomi	Rutaceae	T	Minor bleeding	FrL	F	4	De	3	MA28
				Wound	Fr	F	4	De	5	
*Ruta chalepensis* L.	Tenadam	Rutaceae	H	Stomachache	Fr	F	5	O	4	MA105
				Evil Eyes	L	F	5	O	3	
				Common cold	L	F	5	O	3	
				Malaria	L	DF	1	O	4	
*Citrus medica* L.	Tiringo	Rutaceae	T	Appetite	Fr	DF	5	O	3	MA29
*Osyris lanceolata*Hochst. & Steud	Keret	Santalaceae	S	Rabies	R	F	14	O	2	MA87
				Wound	L	F	2	De	1	
*Dodonaea viscosa*subsp.*angustifolia*(L.f.) J.G.West	Kitkita	Sapindaceae	S	Atopic eczema	L	D	2	De	7	MA41
				Wound	L	DF	2	De	5	
				Eye disease/trachoma	R	D	2	Op	8	
*Verbascum sinaiticum* Benth	Yahya Jero	Scrophulariaceae	H	Febrile disease	L	F	7	Na	11	MA120
				Wound	L	F	2	De	12	
*Physalis lagascae* Roem. & Schult	Awut	Solanaceae	H	Varicella zoster virus	L	F	3	De	2	MA89
				Wound	L	F	3	De	5	
*Nicotiana tabacum* L.	Tinbaho	Solanaceae	S	Evil spirit	L	F	8	Na	3	MA79
				Common cold	L	F	4	Na	4	
*Solanum anguivi* Lam	Zirch-embuay	Solanaceae	H	Toothache	R	D	15	O	4	MA110
*Solanum dasyphyllum*Schumach. & Thonn	Geber-embuay	Solanaceae	S	Coughing	Fr	D	5	O	1	MA135
				Snake bite	R	F	9	O	1	
*Solanum marginatum* L.f	Embuay	Solanaceae	S	Head injury (wound)	L	F	4	De	1	MA113
*Capsicum annuum* L.	Karia	Solanaceae	H	Malaria	Fr	F	9	O	1	MA19
*Datura stramonium* L.	Astenagir/Atsefaris	Solanaceae	S	Dandruff	L	DF	4	De	1	MA39
				Toothache	Se	D	7	De	1	
*Solanum somalense* Franchet	Yeshehochu Kitel/Shejerete Jin	Solanaceae	S	Headache	L	DF	7	Na	2	MA114
				Evil Eyes	L	DF	7	Na	5	
				Febrile disease	L	F	7	Na	2	
				Fever	L	F	7	Na	3	
				Evil spirit	L	DF	7	O	3	
				Swelling	L	DF	3	De	1	
				Toothache	L	D	5	O	2	
				Diarrhea	L	DF	5	O	21	
*Solanum incanum* L.	Embuay	Solanaceae	S	Wound	L	DF	3	De	2	MA112
				Bleeding	L	F	9	O	4	
				Swelling	R	F	3	De	2	
				Stomachache	R	F	11	O	2	
				Bleeding	L	F	7	Na	2	
*Capsicum frutescens* L.	Mitmita Karia	Solanaceae	H	Camel Flue	Fr	D	5	Na	5	MA20
*Solanum lycopersicum *L.	Timatim	Solanaceae	H	Malaria	L	F	11	O	3	MA70
*Withania somnifera* (L.) Dunal	Ede-buda/Gizawa	Solanaceae	S	Headache	R	DF	7	Na	3	MA124
				Evil Eyes	R	DF	7	Na	2	
				Gonorrhea	R	F	1	O	2	
				Syphilis	R	F	1	O	2	
				Febrile disease	L	F	7	Na	2	
				Fever	R	F	7	Na	1	
				Evil spirit	L	DF	7	O	2	
				Swelling	R	DF	3	De	3	
				Toothache	R	D	5	O	9	
*Grewia ferruginea* Hochst. ex A.Rich	Lenkuata	Malvaceae	S	Stomachache	B	DF	11	O	2	MA55
				Asthma	R	DF	11	O	3	
*Grewia villosa* Willd	Agobday	Malvaceae	S	Broken bone	R	DF	3	De	1	MA56
*Grewia bicolor* Juss	Sefa	Malvaceae	S	Stomachache	L	F	9	O	1	MA54
*Celtis africana* Burm.f	Awrarise/Ameleka	Cannabaceae	T	Dactylitis	L	DF	3	De	1	MA25
*Urtica simensis* Hochst. ex A.Rich	Sama	Urticaceae	H	Warts	L	F	4	De	4	MA119
*Verbena officinalis* L.	Atuch	Verbenaceae	H	Stomachache	R	DF	11	O	3	MA121
*Lantana trifolia* L.	Yeregna Kolo	Verbenaceae	S	Eye infection	Fr	D	4	De	1	MA66
*Lippia abyssinica*(Otto & A.Dietr.) Cufod	Kessie	Verbenaceae	S	Stomachache	L	F	9	O	2	MA69
*Lantana camara* L.	Yewef Kolo	Verbenaceae	S	Chronic skin diseases	Fr	F	2	De	1	MA65
*Tapinanthus globifer*(A.Rich.) Tiegh	Yebuna Tegedra	Loranthaceae	H	Diarrhea	L	F	5	O	4	MA123
*Cyphostemma adenocaule* (Steud. ex A.Rich.) Desc. ex Wild & R.B.Drumm	Aserkush Tebetebkush	Vitaceae	C	Syphilis	L	F	2	De	2	MA24
				Herpes zoster	L	F	4	De	1	
*Cissus quadrangularis* L	Yezhon Anjet	Vitaceae	C	Swelling	Wp	F	3	Na	2	MA27
*Zingiber officinale* Roscoe	Zingibil	Zingiberaceae	H	Stomachache	Rh	DF	1	O	7	MA126
				Common cold	Rh	DF	1	O	15	

Key: Growth form, GF (Tree, T; Shrub, S; Herb, H; Climber, C). Ailment treated, AT; Plant part used, PPU (Leaves, L; Root, R; Fruit, Fr, Seed, Se; Flower, Fl; Stem bark, SB; Latex, Lat; Bark, B; Bulb, Bu; Rhizome, Rh; The whole plant part, Wp; Hemiparasite, Hp; Leaves and Root, LR). Conditions of part used, CPU (Dry, D; Fresh, F; Dry and Fresh, DF). Methods of preparation and application, MPAP (1. Boil and drink the decoction when cool; 2. Grind and paint the powder or crushed part; 3. Grind, paste the crushed part and tie; 4. Extract the juice/oil/latex and pour or paint it; 5. Crush, homogenize with cold water and drink; 6. Hold with teeth; 7. Crush, heat/burn or boil the part and inhale its smoke or steam; 8. Crush and sniff the freshly crushed part; 9. Eat the part; 10. Boil and do steam bath; 11. Drink the concoction; 12. Boil the part and paint the decoction; 13. Mixing the plant in local beverage, (Tella); 14. Mixing the plant in milk; 15. Mixing the plant with honey; 16. Mixing the plant with coffee; 17. Cut and drop on the ground). Route of Administration, RA (Oral, O; Dermal, De; Nasal, Na; Optical, Op: Auricular, Au; Anal, An; Oral, Dermal, Nasal, ODN). Use citation, UC; Voucher Specimen, VS

**Table 3:** List of top 15 medicinal plants used for the treatment of human ailments in Habru District based on the use citation report by informants

Scientific name	Use citation	Ailment treated in the study area	Ethnomedicinal use report to treat human ailments elsewhere in Ethiopia
*Solanum somalense* Franchet	39	Headache, evil eyes, febrile disease, fever, evil spirit, swelling, toothache, diarrhea	Typhoid and fire burn [19]
*Ehretia cymosa* Thonn	28	Evil spirit	Headache and abdominal pain [42]
*Cleome gynandra* L.	27	Wound, ear infection, evil spirit	Diarrhea [43]
*Croton macrostachyus* Hochst. ex Delile	26	Atopic eczema, liver problem, stomachache, gonorrhea, malaria, chronic skin diseases, scabies, wound, minor bleeding, febrile disease	Atopic eczema [44], Liver problem, stomachache [45, 46], gonorrhea [18, 45, 47], malaria [46, 47], wound [16, 47], minor bleeding [16], scabies [16, 18, 46], tape worm [45], gastritis [22], bone fracture [22], snake poison [45], wound cancer [13]
*Withania somnifera* (L.) Dunal	26	Headache, evil eyes, gonorrhea, syphilis, febrile disease, fever, evil spirit, swelling, toothache	Asthma/coughing [44, 45], febrile disease [18, 48], typhoid [19], fever [48], swelling [18, 19], cancer [13], malaria [47]
*Eucalyptus globulus* Labill	24	Common cold, coughing, pneumonia, gout, headache, febrile disease	Common cold [17, 18, 22, 49], coughing [17, 22, 44, 46], headache [22], febrile disease [17, 18, 22, 26, 50], influenza [47]
*Ocimum lamiifolium* Hochst. ex. Benth	24	Febrile disease	Febrile disease [15, 17, 26, 46], ear infection [15], coughing [44], headache [47], parasites [25], diarrhea [48]
*Verbascum sinaiticum* Benth	23	Febrile disease, wound	Febrile disease [18], stomachache [18, 45], snake bite [48], tonsillitis [44]
*Zingiber officinale* Roscoe	22	Stomachache, common cold	Coughing [17, 44], stomachache [18, 26, 49, 50], headache [15], common cold [17], influenza [47], cancer [13], malaria [15], swelling [18], tonsillitis [44]
*Allium sativum* L.	21	Atopic eczema, asthma, common cold, dandruff, pneumonia, coughing, malaria	Skin infection [19], febrile disease [22, 45, 51], malaria [17, 18, 22, 45–48], intestinal parasite [48], stomachache [18, 22], influenza [17, 45, 52], common cold [17], headache [15, 52], pneumonia [45], coughing [17], wound [46]
*Dodonaea viscosa* subsp. *angustifolia* (L.f.) J.G.West	20	Atopic eczema, wound, eye disease/trachoma	Diarrhea [22], eye disease/trachoma [18], wound [18, 46, 51], tape worm [45]
*Rumex nervosus* Vahl	20	Warts, wound, fire burn	Stomachache [51], diarrhea [49], scabies [48], wound [18], snake bite [46], breast cancer [13, 46], warts [45]. vomiting [49]
*Euclea racemosa* L	19	Wound	Dandruff [18]
*Olea europaea* L. subsp. *cuspidata* (Wall. & G.Don) Cif	19	Dandruff, stomachache	Asthma [46], psychiatric disease [48], tonsillitis [17, 44], gout [51], dandruff [18], fever [17], tumor [13], eye infection [46, 49], snake bite [19]
*Linum usitatissimum* L.	18	Coughing, stomachache	Asthma [22], liver disease [22], wound [45, 49], placental retention [46], swelling [50]

Among the identified MPs in Habru District, indigenous species hold the predominant position, constituting 107 (79.9%) of the total. These plants demonstrate adaptation to the local ecosystem over time, reflecting their deep-rooted connection to the district. Additionally, the district's plant diversity is enriched by the presence of 23 introduced species (17.2%), offering the potential for therapeutic options. Among the reported MPs, two (1.5%) endemic plant species, *Millettia ferruginea* (Hochst.) Hochst. ex Baker and *Urtica simensis* Hochst. ex A.Rich. were recorded in the study area. These species have IUCN conservation statuses of least concern (LC) and not assessed (NA), respectively.

### Disease types, modes of remedy preparation and application

**Fig. 5:**
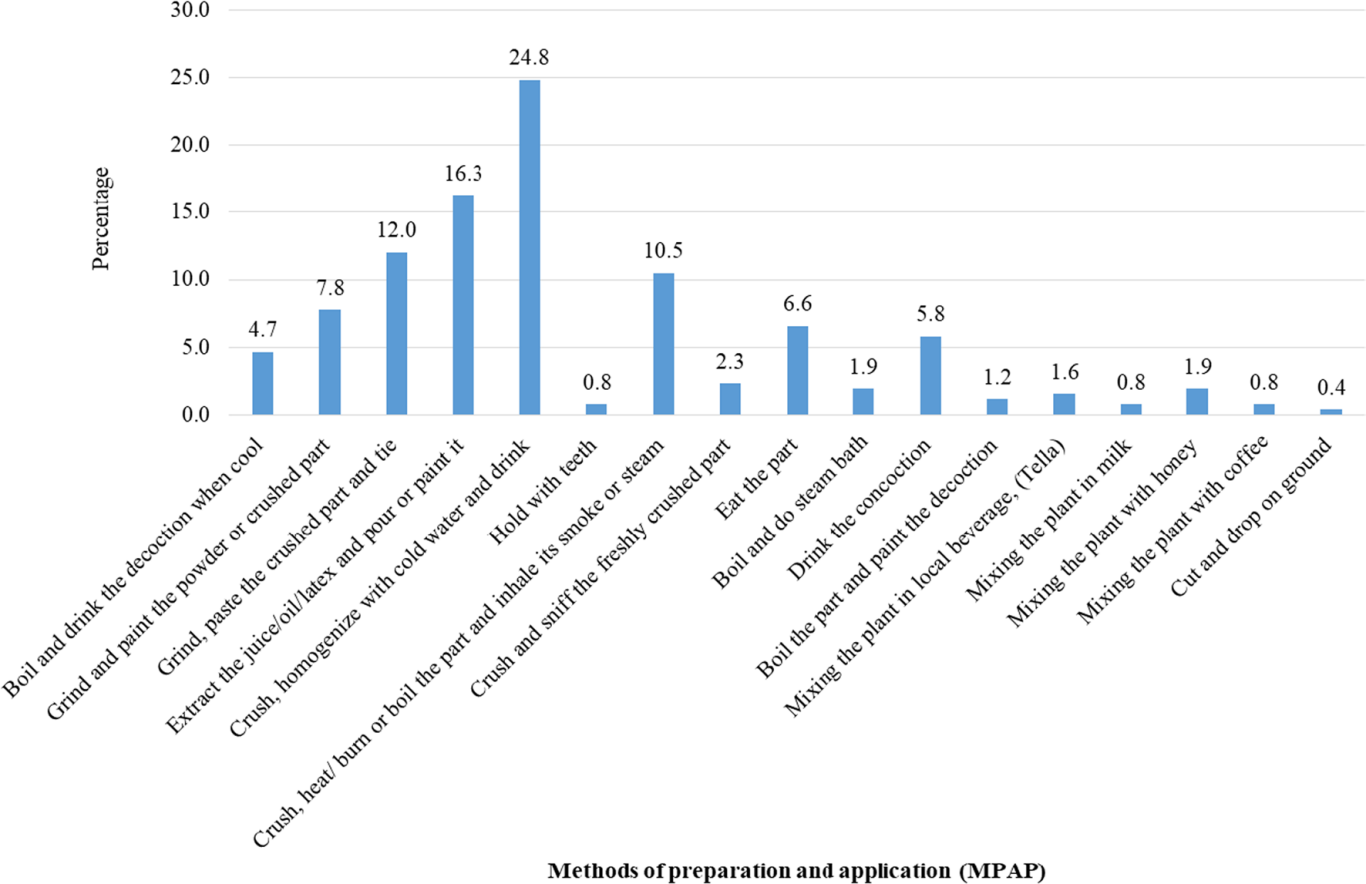
Methods of preparation and application of MPs in the study area

Approximately 50 human health problems were reported in Habru District for which the local people reported being treated with medicinal plants. Wounds, stomachache, and diarrhea were the most commonly reported health problems (30% of the total human ailment reports) under the gastrointestinal disease category, whereas atopic eczema, dandruff, swelling and warts were most frequently reported under the dermatological disease group. The traditional names and clinical explanations of the top ten most cited health problems are indicated in Table 4. The major modes of remedy preparation list were crush, homogenize with cold water and drink (24.8%), extract the juice/oil/latex and pour or paint it (16.3%), grind, paste the crushed part and tie (12%), and crush, heat/burn or boil the part and inhale its smoke or steam (10.5%) (Fig. 5).

**Table 4:** List of the top 10 most cited human health problems in the study area and their clinical descriptions

Local term in Amharic	Clinical term	Clinical descriptions
_KUSIL_	Wound	A disruption to the integrity of biological tissue, including skin, mucous membranes, and organ tissues, caused by various types of trauma [53]
_YEHOD KURTET_	Stomachache	Abdominal pain and/or discomfort can arise from stomach-related issues such as parasites, infections, or allergies [54]
_YEMENFES BESHITA_	Evil spirits	There is no clinical term for "evil spirit". The concept of evil spirits is a religious or spiritual one, not a medical one. In clinical terms, any symptoms or experiences that a person attributes to an evil spirit would be more likely explained by a mental health condition, such as a psychotic disorder, a dissociative disorder, or a seizure disorder [55]
_MECHI/TIKUSAT_	Febrile disease	It is characterized by the presence of fever, which is defined as an elevated body temperature beyond the normal range, usually caused by an infection and resulting from a higher body temperature set point [56]
_GUNFAN_	Common cold	It is an acute, self-limited viral infection of the upper respiratory tract, which may also involve the lower respiratory tract [57]
_TEKMAT_	Diarrhea	Frequent passage of abnormally soft, liquid feces, a symptom of intestinal tract infection caused by a range of bacterial, viral, and parasitic organisms
_SAL_	Coughing	A reflex action that clears the throat and airways of foreign particles, mucus, or other irritants [58]
_YEAYN HIMEM_	Eye Disease/trachoma	A chronic inflammatory disease of the eye and the leading cause of blindness [59]
_FOREFOR_	Dandruff	A scalp condition characterized by the presence of white or grayish flakes of dead skin cells, especially on the scalp [60]
_WEBA_	Malaria	Malaria is a serious and sometimes fatal disease caused by parasites of the Plasmodium group and transmitted to people through the bites of infected female Anopheles mosquitoes [61]

### Plant parts and conditions used

**Fig. 6:**
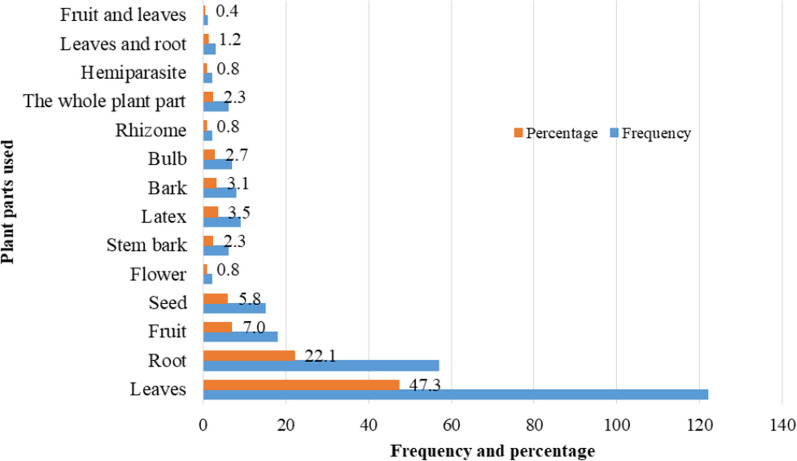
Plant parts used for remedy preparation in Habru District

Plant parts used for remedy preparation indicated that leaves 122 (47.3%) were the widely used plant parts followed by roots 57 (22.1%), fruits 18 (7.0%) and seeds 15 (5.8%) (Fig. 6). The local people of the study area reported employing plant materials of different conditions. The fresh plant materials (parts) were the dominant ones accounting for 58% used in remedy preparation whereas both dried and fresh parts were used at least (24%); the remaining 17% remedies were reported to be prepared from dried parts of medicinal plant species.

### Routes of administration

**Fig. 7:**
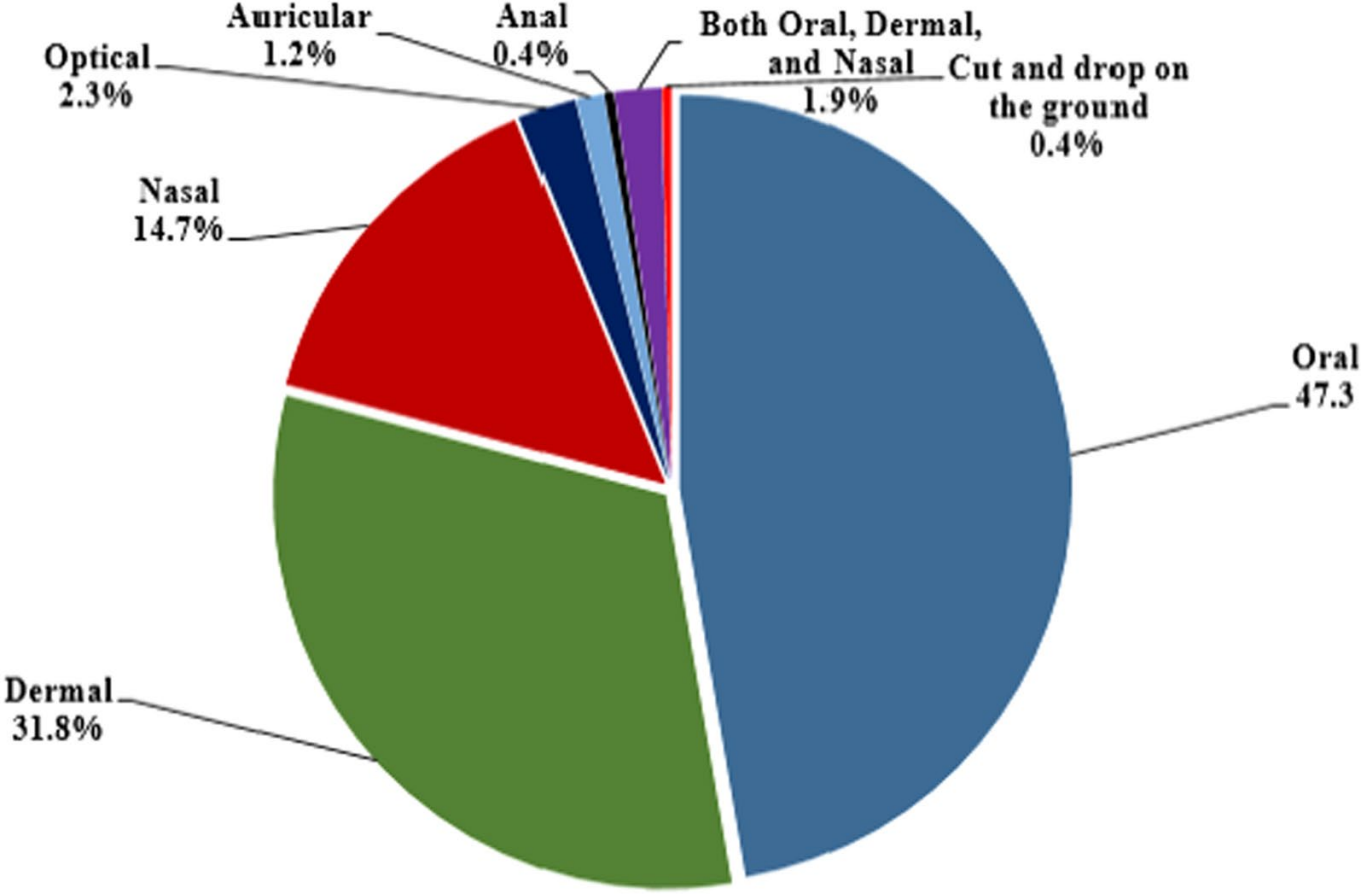
Routes of administration of traditional medicinal plants used in the study area

In Habru District, different methods were used to administer medicinal plant preparations. Accordingly, the major routes of administration in the study area were reported to be oral application (122 preparations, 47.3%) followed by topical or dermal application (82 preparations, 31.8%). The detailes about routes of administration techniques are presented in Fig. 7.

### Marketability of medicinal plants

**Fig. 8:**
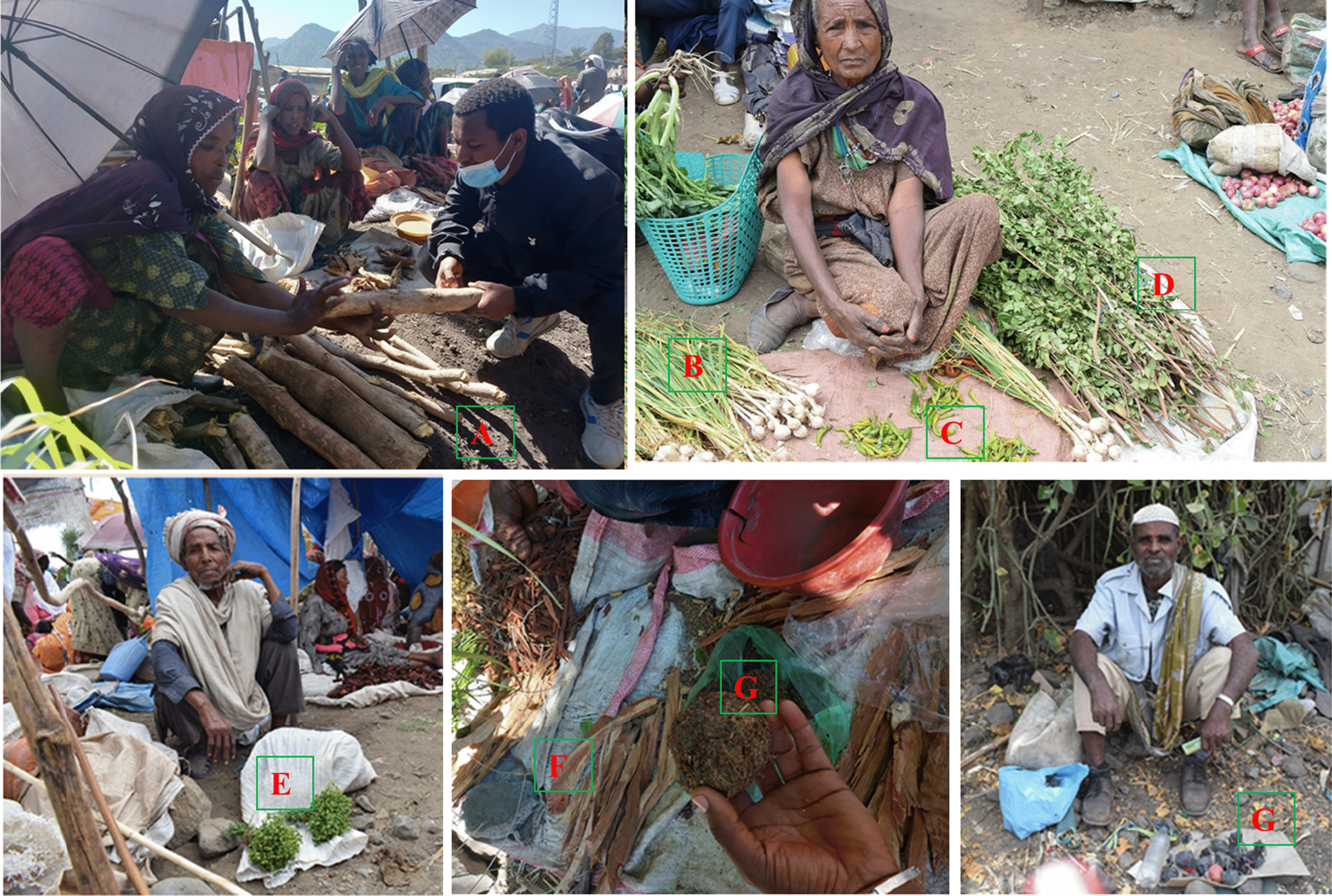
**A** *Terminalia brownii* Fresen. at Girana kebele local market; **B** *Allium sativum* L. and **C** *Capsicum annuum* L.and **D** *Myrtus communis* L. at Mehal Amba kebele local market; **E** *Ruta chalepensis* L., **F** *Olea europaea* L. subsp*. cuspidata* (Wall. & G.Don) Cif., and **G** a bunch of parts of eight processed medicinal plant parts said to treat problems related to wound at Mehal Amba kebele local market. (Photo courtesy: Mulugeta Alemu, Habru District, Ethiopia, 2023)

Among the reported medicinal plants in the study area, 22 (16.4%) species were reported as marketable and only six species (*Terminalia brownii* Fresen., *Myrtus communis* L., *Ruta chalepensis* L., *Olea europaea* L. subsp. *cuspidate* (Wall. & G.Don) Cif., *Allium sativum* L., and *Capsicum annuum* L.) were found in the local markets sold and purchased entirely for their medicinal applications (Fig. 8). The majority of reportedly marketable medicinal plants (82%) were mainly sold for their non-medicinal uses but occasionally applied as medicine when the need arises. The average price of 25–40 cm long and 10 cm diameter *Terminalia brownii* Fresen. (WEYBA) stem at the Girana local market was 40 Birr (0.75 USD), whereas for a bunch (300–400 gm) of the branch material of *Myrtus communis* L. (ADES) was 10 Birr (0.21 USD); and the price was 15 Birr (0.28 USD) for a bunch (100–150 gm) of *Ruta chalepensis* L. (TENADAM) branch and fruit. A coffee cup of eight types of medicinal plant powder was sold for 15–20 Birr (0.28–0.37 USD) at Mehal Amba local market for treating dandruff, in which the seller was not interested in mentioning the name of these plants.

### Efficacy of medicinal plants

From the entirety of 50 distinct human ailments reported within Habru District, nine disease categories were identified (Table 5). Among these categories, those exhibiting the highest informant consensus factor (ICF) values were gastrointestinal and parasitic ailments (0.85), followed closely by febrile diseases (0.84), cultural-related conditions such as evil spirits and evil eyes (0.84), and throat and respiratory diseases (0.80).

**Table 5:** ICF values of traditional medicinal plants for treating human ailments in Habru district

No	Disease category (DC)	Reported diseases	Species	% of all species	Use citation	% of all use citation	ICF value
1	Gastrointestinal and parasitic	Stomachache disease, tap worm, typhoid, giardia, gastritis, diarrhea, abdominal pain, constipation, intestinal parasite, vomiting	23	18.0	151	19.48	**0.85**
2	Dermatological	Atopic eczema, dandruff, scabies, wounds, chronic skin diseases, over blood flow after birth, excessive bleeding after birth, fire burn, minor bleeding, warts, swelling, varicella zoster virus, taking out the spine, boils	46	35.9	220	28.39	0.79
3	Throat and Respiratory Diseases	Tonsillitis, asthma, Common cold, Coughing, pneumonia	24	18.8	117	15.10	0.80
4	Febrile disease	Febrile disease, fever	11	8.6	63	8.13	**0.84**
5	Animals and insects cause poisonous diseases	Rabies, snake bite, Malaria	17	13.3	36	4.65	0.54
6	Evil spirits	Evil spirits, evil eyes	14	10.9	80	10.32	**0.84**
7	Organ diseases	Toothache, ear infection, ear mites, eye disease/trachoma, liver problems, jaundices	20	15.6	65	8.39	0.70
8	Musculoskeletal (Bone fracture	Bone fracture	3	2.3	6	0.77	0.60
9	Others	Syphilis, gingivitis, gout, headache, hemorrhoid, impotency, gonorrhea	15	11.7	44	5.68	0.67

Bold values represent the highest ICF value within each disease category

### Relative healing potential of medicinal plants

The highest fidelity level (91.3%) was recorded for *S. somalense* followed by *O. lamiifolium* (88.9%) and *V. sinaiticum* (85.7%) (Table 6). The recorded highest fidelity level values of *S. somalense* and *O. lamiifolium* were obtained under the gastrointestinal and parasitic and febrile therapeutic categories, respectively.

**Table 6:** Fidelity level value of ten medicinal plants commonly reported for use against a given ailment category

No	Medicinal plant species	Therapeutic category	Np	N	FL value (%)	Rank
1	*Solanum somalense* Franchet	Diarrhea	21	23	91.3	1
2	*Ocimum lamiifolium* Hochst. ex. Benth	Febrile disease	24	27	88.9	2
3	*Verbascum sinaiticum* Benth	Wound	12	14	85.7	3
4	*Withania somnifera* (L.) Dunal	Toothache	9	11	81.8	4
5	*Calpurnia aurea* (Ait.) Benth	Diarrhea	12	16	75.0	5
6	*Rumex nervosus* Vahl	Wound	13	18	72.2	6
7	*Linum usitatissimum* L.	Stomachache	13	19	68.4	7
8	*Zingiber officinale* Roscoe	Common cold	15	22	68.2	8
9	*Euclea racemosa* L.	Wound	19	28	67.9	9
10	*Olea europaea* L. subsp. *cuspidata* (Wall. & G.Don) Cif	Dandruff	12	19	63.2	10

Where FL = Fidelity Level, Np = number of informants who independently cited the importance of a species for treating a particular disease; N = total number of informants who reported the plant for any given disease

### Use diversity of medicinal plants

The results obtained from the direct matrix ranking (DMR) exercise conducted on nine multipurpose medicinal plants enabled the identification of the specific plant facing the highest pressure within the area, along with the associated factors responsible for posing a threat to the plant. Accordingly, the DMR showed that *O. europaea* subsp. *cuspidata* ranked first (most threatened) followed by *D. angustifolia* and *E. racemosa* (Table 7).

**Table 7:** Average DMR score of ten key informants for nine medicinal plant species with additional uses

Use diversity	Multipurpose medicinal plants subjected to DMR									Total	Rank
	*Searsia retinorrhoea* (Steud. ex Oliv.) Moffett	*Ehretia cymosa* Thonn	*Juniperus procera* Hochst. ex Endl	*Euclea racemosa* L	*Croton macrostachyus* Hochst. ex Delile	*Eucalyptus globulus* Labill	*O. europaea* L*.* subsp*. Cuspidata* (Wall. & G.Don) Cif	*Rumex nervosus* Vahl	*Dodonaea angustifolia* subsp. *angustifolia* (L.f.) J.G.West		
A-Agricultural tools	5	4	2	3	2	2	4	0	4	26	4
B-House construction	4	3	5	5	3	5	5	1	5	36	**1**
C-Firewood	3	4	3	5	5	3	4	2	5	34	**2**
D-Fodder	0	1	0	1	0	0	0	3	1	6	6
E-Medicine	2	5	3	3	4	5	2	5	3	32	3
F-Charcoal	1	0	3	2	0	0	5	0	1	12	5
Total	15	17	16	19	14	15	20	11	19		
Rank	6	4	5	**2**	8	6	**1**	9	**2**		

Bold values indicate the highest-ranked use for each medicinal plant

Based on use criteria (5 = excellent; 4 = very good; 3 = good; 2 = less used; 1 = least used and 0 = no value)

### Use of wild medicinal plants as awild edibles in the study area

In the study area, some plants have dual roles being used both as medicinal and wild edible resources. From the total reported medicinal plants, 11 species (8.2%) were identified as being utilized as wild edible plants in the study area. Among these plants, shrubs constituted 55%, while trees accounted for the remaining 45% in terms of growth habit. The edible plant parts utilized were diverse, with 82% of them being fruits, 9% comprising the inner parts of stems, leaves, and tender shoots, and 9% involving leaves, stems, and bark. In addition, the method of preparation varied with 82% of the plants featuring ripe fruit was consumed raw (Table 8). An additional 9% encompassed young tender shoots, leaves, and the inner part of stems, which are consumed fresh and raw. A distinct 9% of plants were found to be employed for flavoring traditional alcoholic beverages such as Tela (made from honey) and Tej (made from fermented grains).

**Table 8:** List of medicinal plants used as wild edible plants in Habru District

Scientific names	Local Name	Family names	Growth habit	Edible plant part	Method of preparation
*Carissa spinarum* L.	Agam	Apocynaceae	Shrub	Fruit	Ripe fruit eaten raw
*Cordia africana* Lam	Wanza	Boraginaceae	Tree	Fruit	Ripe fruit eaten raw
*Opuntia ficus-indica* (L.) Mill	Beles, Qulqual	Cactaceae	Shrub	Fruit	Ripe fruit eaten raw
*Euclea racemosa* L.	Dedeho	Ebenaceae	Shrub	Fruit	Ripe fruit eaten raw
*Ficus sur* Forssk	Sholla	Moraceae	Tree	Fruit	Ripe fruit eaten raw
*Ficus vasta* Forssk	Warka	Moraceae	Tree	Fruit	Ripe fruit eaten raw
*Rumex nervosus* Vahl	Embacho	Polygonaceae	Perenial herb	Inner part of stem, leaves and tender shoots	Young tender shoots, leaves and inner part of stem eaten fresh & raw
*Ziziphus spina-christi* Desf	Kurkura, Geba	Rhamnaceae	Tree	Fruit	Ripe fruit eaten raw
*Rhamnus prinoides* L'Hér	Gesho	Rhamnaceae	Shrub	Leave, Stem and bark	To flavor traditional alcoholic drinks (Tela and Tej)
*Rosa abyssinica* R.Br. ex Lindl	Kega	Rosaceae	Shrub	Fruit	Ripe fruit eaten raw
*Citrus aurantiifolia* (Christm.) Swingle	Lomi	Rutaceae	Tree	Fruit	Ripe fruit eaten raw

### Preference ranking

A preference ranking exercise with 15 key informants on medicinal plants that were reported to be used against diarrheal diseases, the most frequently reported disease in the gastrointestinal and parasitic disease category, showed that *S. somalense* (yeshehochu kitel), *C. aurea* and *V. sinaiticum* (yeahiya joro) were the most preferred species to treat the reported disease (Table 9).

**Table 9:** Results of preference ranking of seven medicinal plants reported for treating diarrheal diseases

Medicinal plants reported for treating diarrheal diseases	Local Name in Amharic	Family name	Informants labeled KI1 to KI15															Total score	Rank
			KI1	KI2	KI3	KI4	KI5	KI6	KI7	KI8	KI9	KI10	KI11	KI12	KI13	KI14	KI15		
*Barleria eranthemoides*	Yeset Af	Acanthaceae	1	3	3	1	2	1	2	1	4	5	3	3	4	8	7	48	8
*Justicia schimperiana*	Sensel	Acanthaceae	2	2	5	3	1	4	4	2	5	2	6	1	2	7	1	47	9
*Carissa spinarum*	Agam	Apocynaceae	4	1	1	5	6	6	1	4	3	1	2	6	3	2	5	50	7
*Calpurnia aurea*	Digita	Fabaceae	9	10	7	9	8	10	9	10	9	8	5	10	10	9	10	133	2
*Olea europaea* subsp. *cuspidata*	Weira	Oleaceae	3	5	2	6	4	2	5	3	1	4	1	2	1	3	2	44	10
*Ruta chalepensis*	Tenadam	Rutaceae	6	8	4	4	5	3	3	7	6	3	7	5	8	5	3	77	5
*Verbascum sinaiticum*	Yahya Jero	Scrophulariaceae	7	6	9	8	10	7	8	8	8	9	8	7	6	4	8	113	3
*Withania somnifera*	Ede-buda/Gizawa	Solanaceae	8	7	8	7	7	8	7	6	7	7	10	9	5	6	6	108	4
*Solanum somalense*	Yeshehochu Kitel	Solanaceae	10	9	10	10	9	9	10	9	10	10	9	8	9	10	9	141	1
*Rumex nervosus*	Embacho	Polygonaceae	5	4	6	2	3	5	6	5	2	6	4	4	7	1	4	64	6

NB: Scores in the table indicate ranks given to medicinal plants based on their efficacy. The most important in the set is given the highest number, decreasing in number as the members of the set decrease in importance. This implies the highest number (10) given for the medicinal plant which informants thought most effective in treating evil spirits and the lowest number (1) for the least effective plant

### Traditional herbal medicine: opportunities, challenges, and indigenous conservation practices in Habru District

The participants in the FGD identified several opportunities for the utilization, regulation, and promotion of traditional medicine in the district. They emphasized the importance of raising awareness about traditional medicine (TM) and forest management practices. Additionally, the participants highlighted the need to strengthen research and development activities to further enhance the utilization of TM. They also expressed the potential for scaling up TM utilization by providing support through research-based inputs. Furthermore, the participants recognized the value of supporting traditional healers through training and material assistance. Continuous supervision from the Ministry of Health and other responsible organizations was seen as an opportunity to ensure the effective implementation of the study.

The FGD highlighted various challenges and threats that impact the utilization, regulation, and promotion of traditional medicine in the kebele. Among these, participants highlighted the detrimental effects of deforestation, agricultural expansion, firewood collection, and environmental degradation on medicinal plants. According to the responses from key informants, these factors collectively pose significant threats to the availability and sustainability of medicinal plants in the study area. The loss of habitat due to deforestation and agricultural activities, coupled with the increasing demand for firewood, has emerged as a prominent concern, potentially leading to the depletion of essential plant species used in traditional medicine. Furthermore, environmental degradation further exacerbates these threats, underscoring the urgent need for conservation efforts to safeguard valuable medicinal plant resources.

### Indigenous knowledge on conservation practices

In this study, about 91.7% of the FGDs (11 out of 12) mentioned the cultivation of medicinal plants within home gardens; all FGDs (12 out of 12) stressed the importance of guarding against deforestation and fire; about 91.7% of the FGDs (11 out of 12) emphasized the need to control the massive harvest of wild medicinal plants; and finally, 100% of the FGDs (12 out of 12) highlighted the practice of maintaining seedlings in nurseries, planting, and overall conservation efforts as vital methods for safeguarding medicinal plant species.

To address the identified challenges and leverage the opportunities, the FGD participants put forth several recommendations. They called for intensified awareness campaigns about traditional medicine and forest management practices. Strengthening research and development activities emerged as a key recommendation to enhance the effectiveness of traditional medicine utilization. The participants also recommended scaling up the utilization of traditional medicine through research-based inputs and by offering support to traditional healers through training and material assistance. The need for continuous supervision from the Ministry of Health and other responsible organizations was underscored to ensure proper regulation and promotion of traditional medicine practices. Additionally, the participants emphasized the importance of addressing the issue of invasive plant species by implementing measures to replace them with native plants, thereby contributing to the conservation of plant diversity and the facilitation of traditional medicine utilization in the study kebeles.

## Discussion

The ethnobotanical studies conducted in Ethiopia have revealed a rich and diverse knowledge of medicinal plants among the country's various ethnolinguistic groups [22, 62]. In Ethiopia, approximately 800 plant species are traditionally used to treat various human and livestock ailments [63, 64]. The results of the present study are consistent with these findings, as they document the use of a wide variety of medicinal plants to treat a wide range of ailments in the country. The findings of this study underline the diversity and use of reported medicinal plants in Habru District. The taxa documented in this study (134 species belonging to 110 genera and 54 families) reflect the depth and breadth of the medicinal plants both taxonomically and in their medicinal lore as well as the functional attributes of each species. The list of medicinal plant species identified through ethnobotanical surveys highlights the rich botanical heritage of the area. The diversity of medicinal plants documented in the Habru District aligns with the studies conducted in adjacent areas. In Tenta District, South Wollo, Ethiopia, a total of 121 medicinal plant species were identified, with Fabaceae dominating the list [23] and the trend is comparable to the Habru District's botanical landscape. Similarly, the study conducted in Gubalafto District, which neighbors the study area, 135 traditional medicinal plant species were documented, with Asteraceae being notably abundant [18], aligning with the Habru District's diversity. Moreover, in Yalo Woreda, Afar regional state, Ethiopia, 106 medicinal plants were reported, emphasizing the prevalence of Fabaceae [19], a finding consistent with the Habru District and surrounding areas. These similar findings across different regions illustrate the prevalence and importance of specific plant families, underscoring the collective richness of medicinal plant diversity in these geographically adjacent areas. For example, this study identified species such as *R. chalepensis*, *O. europaea* L. subsp. *cuspidata*, *A. sativum*, *C. annuum*, *O. lamiifolium*, and *V. sinaiticum*, aligning with findings from studies conducted in Gubalafto, Tenta, and Yalo Districts. Additionally, frequently cited medicinal plants were mentioned in the study area also found in another region of Ethiopia such as *S. somalense* [19], *E. cymosa* [42], *C. gynandra* [43], *C. macrostachyus* [18, 22, 45, 47] and *W. somnifera* [13, 18, 19, 44, 45, 48] which was in line with study in the neighboring regions. This may be attributed to the similar ecological landscape and cultural attributes of the neighboring regions.

Moreover, several studies in Ethiopia have reported Solanaceae as the most dominant medicinal plant family [14, 17, 24, 46, 51, 65–67]. These studies showed that members of the Solanaceae family are renowned for their medicinal attributes and contain many phytochemicals that offer potential health benefits. Notable phytochemicals within this family encompass glycoalkaloids, anthocyanins, alkaloids, flavonoids, and terpenoids, as extensively documented [68, 69]. One study conducted in Seharti Samre District, Southern Tigray, Ethiopia found Solanaceae, Lamiaceae, and Fabaceae as the highest contributors of medicinal plants [46]. Another study conducted in Damot Woyde District, Wolaita Zone, Southern Ethiopia, found that Solanaceae was represented by 5 species, making it the third most common family of medicinal plants [17]. Similarly, a study conducted in Ada'a District, East Shewa Zone of Oromia Regional State, Ethiopia, found Solanaceae to be one of the leading plant families that encompass large medicinal species [51]. The use of medicinal plants in the study area revealed a notable distribution, with the majority of species categorized as herbs (36%), followed by shrubs (32%) and trees (25%). This prevalence of herbs could be attributed to their proximity and ease of accessibility in nearby areas compared to trees and shrubs which are often harvested from more remote patches of forested areas. This finding agrees with the pattern of dominance of herbaceous species both in Ethiopia and other countries [15, 16, 52, 70, 71].

Ethiopia is home to several endemic plant species, some of which are used for medicinal purposes. A review of Ethiopian endemic plants identified a total of 44 endemic medicinal plant species belonging to 20 families and 30 genera [72, 73]. In this study, the identification of two endemic medicinal plant species (*M. ferruginea* and *U. simensis*) in Habru District further emphasizes the unique ecological and botanical characteristics of the study district. These endemic species hold immense value in terms of their potential therapeutic properties and may contribute to the broader field of ethnopharmacology. The presence of such endemic medicinal plants signifies the distinctiveness of the local flora and its role in traditional healing practices.

The disease types identified, along with the diverse modes of remedy preparation and application, reflect the intricate traditional knowledge of the local community. Results revealed that gastrointestinal diseases and dermatological diseases are common health problems in the study area. Similar results were reported by [16]; constipation and diarrhea are some of the most commonly reported health problems under the gastrointestinal disease category in Ankober District, North Shewa Zone, Amhara Region, Ethiopia [16], whereas [74] reported that intestinal parasitic infections are common among prison inmates in Ethiopia.

Traditional healers in the study area stated that they prepare remedies using a variety of methods, depending on the type of illness and the corresponding explanations by their patients. The utilization of different plant parts, often specific to certain conditions, showcases the careful selection and application of plant resources based on their perceived effectiveness. According to several studies on Ethiopian medicinal plants [14, 25, 47, 73, 75], leaves are the most commonly used plant parts for remedy preparation, followed by roots and other plant parts such as seeds, stems, bark, fruits, young shoots, and flowers. In this study also leaves (122, 47.3%) cases are the most widely used plant part followed by roots. This is because these plant parts play a vital role in the whole life cycle of the plant species as they are the sites of various metabolic reactions and centers where high concentrations of secondary metabolites are found.

In the present study, fresh plant parts were the dominant ones (58.2%) used in remedy preparation against various human ailments. The use of freshly harvested plant parts is believed to enhance the efficacy of the remedies, as they are considered to contain higher levels of active ingredients of fresh plant parts that could be lost on drying. Similar findings were reported by [6, 15, 16, 25].

Routes of administration represent another dimension of traditional medicine, with various methods employed to harness the therapeutic benefits of medicinal plants. The study reveals the multifaceted ways in which these plants are integrated into local healthcare practices, ranging from oral ingestion to external applications. This diversity in routes of administration contributes to the versatility of traditional medicine. Accordingly, the major routes of administration in the study area were reported to be oral application (47.3%). Similar findings were reported [16, 18, 25] stating that the oral route is the most common route of administration for medicinal plant preparations in Ethiopia. It involves consuming the remedies orally, either in the form of powders, decoctions, or infusions.

The results from the market survey of medicinal plants indicated that only 16.4% of species were reported as marketable and the remaining 82.8% had no marketability report since they were not available in the local market of the district during the time of the research. Accordingly, *T. brownii*, *M. communis*, *R. chalepensis*, *O. europaea* subsp. *cuspidata*, *A. sativum* L., and *C. annum* were found on the local markets sold and purchased entirely for their medicinal applications. This indicates a good market demand for these plants, potentially leading to important economic returns for local communities involved in their conservation, trade, and utilization of MPs.

The highest recorded ICF values (0.85 and 0.84) indicated the best agreement among informants on the use of medicinal plant species reported to be used for treating gastrointestinal and parasitic ailments and febrile diseases, respectively. The efficacy of medicinal plants, closely tied to traditional healing practices, holds cultural and practical significance. The recognition of certain plants as highly efficacious underscores the importance of preserving and further exploring traditional knowledge for future healthcare advancements. The highest ICF values are important to identify plants of particular interest in the search for bioactive compounds [40]. Accordingly, a total of 23 medicinal plants of Habru District (with high ICF values) for treating gastrointestinal and parasitic diseases are under investigation for their pharmacological properties by our research theme.

Fidelity level is a measure of the consistency of a plant's use for a particular ailment or purpose across different cultures or regions [40]. The finding in this study of the highest FL values for *S. somalense* (91.3%) against gastrointestinal and parasitic diseases and *O. lamiifolium* (88.9%) against febrile diseases; and *V. sinaiticum* (85.7%) against wound could be considered the relative healing potential of medicinal plants against the corresponding diseases and provide valuable insights into local perceptions of efficacy and potency. Plants with the highest fidelity level values could also be targeted for further phytochemical investigation to prove the bioactive components and conservation efforts, as they may have important medicinal or cultural significance [76]. Accordingly, further activity testing experiments are being carried out on extracts of these species by our research group. This understanding shapes preference ranking and contributes to the prioritization of certain plant species in healthcare practices.

The output of a DMR exercise showed the highest ranks for *O. europaea* subsp. *cuspidata*, *D. angustifolia* and *E. racemosa,* which are the multipurpose plant species [17] of the area based on analysis of the information collected from key informants.. This result indicates that these plants are exploited more for their non-medicinal uses (house construction and firewood) than for reported medicinal values. Overharvesting of multipurpose medicinal plant species for house construction and firewood was found to be the responsible factor aggravating the depletion of the species in the area. Thus, the study findings highlight the need for immediate complementary conservation measures to save the fast-eroding multipurpose medicinal plant species in the study area. Research results also reported that multipurpose medicinal plant species are often overexploited for purposes other than their medicinal value, such as fuel wood, charcoal production, construction materials, and lumbering [16, 17, 26, 71, 77].

The interconnection between medicinal and edible plants has been recognized across various cultures and regions. The utilization of medicinal plants as wild edible plants presents an opportunity for communities to benefit from their nutraceutical role [78]. Similarly, a study in South Africa found that several wild edible vegetables were used for medicinal purposes [79]. Overall, the utilization of medicinal plants as wild edible plants in Ethiopia contributes to food security, provides nutritional benefits, and showcases the rich traditional knowledge of local communities. Further research is needed to explore the nutritional and medicinal properties of specific plant species and their potential for sustainable food systems [27, 80, 81]. The preference ranking exercise also helped to identify the most preferred medicinal plant species to treat diarrheal diseases under the gastrointestinal and parasitic disease categories. Accordingly, *S. somalense* (yeshehochu kitel), *C. aurea* (Digita.) and *V. sinaiticum* (yeahiya joro) scored the highest values indicating that they are the most preferred species to treat diarrheal diseases in the study area.

Generally, this study of traditional medicinal plants in Habru District revealed the opportunities and challenges facing the plant and knowledge resources. The opportunities identified by the local community, including raising awareness, strengthening research and development activities, and supporting traditional healers, indicate a potential path toward the enhanced utilization and regulation of herbal medicine. However, challenges such as deforestation, agricultural expansion, and environmental degradation pose significant threats to medicinal plant resources, necessitating focused conservation efforts. The findings of this study indicated the intricate interplay among the biodiversity, cultural practices, and healthcare systems in Habru District. The diverse array of medicinal plants, along with their traditional applications, provides a valuable foundation for further exploration, conservation, and potential integration into modern healthcare practices.

According to the comparison of our findings with other ethnobotanical study in Ethiopia, novel plant uses of some medicinal plants were documented. *S. somalense* was used to treat headache, febrile disease, fever, swelling, toothache, and diarrhea, whereas, *C. gynandra* for treating wound and ear infection was completely novel use in the study area and never ever reported. In general, the documented medicinal plant species and associated knowledge have the potential to contribute for the future public health initiatives and the development of sustainable herbal medicine practices in Habru District. The findings of this study can be used to inform the development of culturally sensitive public health interventions, empower local communities to manage their own health needs, and guide future research on the therapeutic properties of the identified plants.

## Conclusions

This study indicated the relationship between the local community and the diverse array of medicinal plant species in Habru District. Traditional medicinal plant species are potential sources in the primary traditional healthcare systems of the people in the study area. The identification of 134 plant species, including 2 endemics, underscores the area's unique biodiversity and its role in conserving useful plants. The participation of informants from diverse demographics has enriched our understanding, revealing variations in ethnobotanical knowledge across gender, age groups, literacy levels, and marital status. The study relied on triangulated data collection and analysis techniques considering both emic and etic perspectives and using both qualitative and quantitative approaches. Such approaches enhance the validity of our findings and reveals of the depth of the community's practices and the key plant species that capture researchers’ attentions. The documentation of MPs in terms of use value (UV), PR scores, and FL values would empower the potential to strengthen future pharmaceutical and phytochemical explorations, as well as conservation initiatives. Consequently, it becomes imperative to focus on safeguarding the traditional medicinal plants and the associated indigenous knowledge within the study are and beyond, ensuring their sustainable use and continuity into the future. These findings serve as valuable resources for sustainable conservation strategies, healthcare practices, and the preservation of traditional knowledge, underscoring the intricate interdependence of human societies and their natural surroundings.

## Recommendations

Traditional healers and the local people who use medicinal plants in Habru District would need support from the education sector, tailored training, and finance to gain better knowledge of medicinal plant conservation and improve the mode of sustainable utilization. Further research needs to be conducted on antimicrobial, antioxidant and phytochemical profiling of potentially effective medicinal plants used in the study area, priority being given to *S. somalense*, *V. sinaiticum*, *R. nervosus, W. somnifera* and *C. aurea,* all of which are used against diarrheal diseases, found to be common in the study area and elsewhere in Ethiopia and beyond to be used as an input for future pharmacological research and development.

## Data Availability

The authors declare that all other data supporting the findings of this study are available within the article and its supplementary information files.

## Abbreviations

AAU: Addis Ababa University
AHRI: Armauer Hansen Research Institute
ArcGIS: Aeronautical Reconnaissance Coverage Geographic Information System
DMR: Direct Matrix Ranking
DPBBM: Department of Plant Biology and Biodiversity Management
ENMA: Ethiopian National Meteorology Agency
ETH: Ethiopia
FGD: Focus Group Discussion
FL: Fidelity Level
HHs: Households
ICF: Informants’ Consensus Factor
IUCN: The International Union for Conservation of Nature
MAPA: Methods of Preparation and Application
PR: Preference Ranking
TM: Traditional Medicines
UV: Use Value
